# Primary health care reforms: a scoping review

**DOI:** 10.1017/S1463423625000271

**Published:** 2025-08-18

**Authors:** Ahmad Shirjang, Leila Doshmangir, Mohammad Bazyar, Vladimir Sergeevich Gordeev

**Affiliations:** 1 National Center for Health Insurance Research, Iran Health Insurance Organization, Tehran, Iran; 2 Department of Health Policy & Management, Health Services Management Research Center, School of Management and Medical Informatics, Tabriz University of Medical Sciences, Tabriz, Iran; 3 Social Determinants of Health Research Center, Health Management and Safety Promotion Research Institute, Tabriz University of Medical Sciences, Tabriz, Iran; 4 Health and Environment Research Center, Ilam University of Medical Sciences, Ilam, Iran; 5 Department of Health Management and Economics, Faculty of Health, Ilam University of Medical Sciences, Ilam, Iran; 6 Department of Infectious Disease Epidemiology, London School of Hygiene & Tropical Medicine, London, UK

**Keywords:** health policy and systems research, health reform, primary health care, public health

## Abstract

**Background:**

Demographic transitions, societal changes, and evolving population health needs are placing increasing pressure on healthcare systems, necessitating ongoing reforms. Primary health care (PHC) is a foundational component of Universal Health Coverage (UHC) and sustainable health systems. Many countries have undertaken PHC reforms aimed at improving population health. This review explores the objectives, implementation mechanisms, challenges, and outcomes of these reforms.

**Methods:**

We conducted a systematic review of studies sourced from five databases (PubMed, Scopus, Proquest, Embase, and Science Direct), applying the World Health Organization’s Health Systems Framework for deductive content analysis. The PRISMA guidelines were followed to ensure transparency and rigour in summarizing the published literature.

**Results:**

A total of 147 types of interventions were identified, with most targeting service delivery and financing. Key reform objectives included expanding access to care, improving financing and payment systems, scaling up family physician programmes, increasing government health expenditure, leveraging private sector capacities, and strengthening the PHC workforce. These interventions resulted in expanded public health coverage, enhanced access to PHC, increased utilization of services among low-income populations, broader social insurance coverage, and improved service quality, contributing to better community health outcomes.

**Conclusion:**

The success of PHC reforms depends on their alignment with political, social, and cultural contexts, as well as consideration of the social determinants of health. Strong governmental support, managerial stability, decentralization, and regional capacity building are essential for sustainable implementation. Reforms should be gradual, supported by accurate forecasting, adequate and sustainable resources, and evidence-based strategies, drawing on international experiences.

## Introduction

Primary health care (PHC) is built on the fundamental premise that individuals, families, and communities have the right to better healthcare. It provides a whole-of-society approach to health and well-being centred on the needs and preferences of all (Allen 2000, Doshmangir, Moshiri et al. 2019). This notion rendered PHC an essential component of healthcare delivery, encompassing health promotion, disease prevention, treatment, rehabilitation and palliative care. PHC represents the first level of care committed to promoting social justice principles, inter-sectoral cooperation, and public participation (Bagyani-Mogadam and Ehraampoosh 2003). These principles are well outlined in several global public health milestones and considered the most efficient and effective ways to achieve health for all (Malekafzali 2014).

By fortifying primary healthcare, the reform can foster a transition from a predominantly remedial model to a preventive and promotive one, with the objective of ameliorating overall health outcomes and diminishing healthcare expenditures (Barnes *et al.*, 2016). Consequently, primary healthcare reform assumes a pivotal role in engendering a more sustainable and patient-centric healthcare system (Majid and Wasim 2020).

In the interim, alongside the function of governmental providers, the function of private providers is of utmost significance, particularly in the latest reforms pertaining to primary healthcare (Sanadgol, Doshmangir *et al.* 2021, Sanadgol, Doshmangir *et al.* 2022, Sanadgol, Doshmangir *et al.* 2022). In the same direction, the Astana Summit in 2018 reiterated the need for continuous action from governments, global health leaders and development institutions, non-governmental organizations, academia and other professional organizations to improve PHC services (Wass 2018). For decades, countries implemented major PHC reforms to meet the changing population’s health needs and expectations. For example, some countries (e.g., Australia, Iran, and the United Kingdom) experienced an improvement in many health indicators following the scaling-up of PHC-based interventions (Saltman and Figueras 1998, Asaei 2014). However, the World Health Organization’s (WHO) reports indicated that most health systems worldwide are still underperforming and recommended establishing health systems based on the critical principles of PHC (Malekafzali 2014, Auener *et al.*, 2020). Several studies have shown a positive relationship between strengthening the PHC system and socio-economic development, which could serve as the basis for the national health system strengthening (Doshmangir *et al.*, 2019, Pinto *et al.*, 2019, Doshmangir *et al.*, 2020) and progress towards Universal Health Coverage (UHC) (Faye, Bob et al. 2012, Organization 2018). Therefore, over the last 20 years, most reforms have expanded and improved PHC services (WHO 2000).

Due to the complex nature of PHC system reforms, understanding countries experiences could help when choosing appropriate policy interventions in PHC systems, facilitating the movement towards UHC, and fostering the 2030 Sustainable Development Goals Agenda. Therefore, this study explored PHC system reform interventions, their aims and achievements through the lens of the primary building blocks of the WHO Health System Framework.

## Methods

Our scoping review adhered to the Preferred Reporting Items for Systematic Reviews and Meta-Analyses protocol, specifically the extension for Scoping Reviews (PRISMA-ScR) as illustrated in Appendix S1 (Tricco *et al.*, 2018), to the goal of answering the main research question ‘the aims of PHC reforms, their mechanisms of implementation, and their achievements’. The intent of the PRISMA-ScR is to facilitate comprehension among a diverse range of readers, by providing a comprehensive overview of pertinent terminology, fundamental principles, and crucial elements that are essential for conducting scoping reviews (Tricco *et al.*, 2018).

### Inclusion and exclusion criteria

We identified and reviewed the literature on PHC reforms, their dimensions, related interventions, and PHC reforms effectiveness in moving towards UHC across countries. We limited our search to papers published in English from January 1990 to January 2023, and our review included summaries, posters, letters to the editor, reviews, commentaries, and opinion pieces.

### Data sources and search

We systematically searched for primary and secondary studies using five databases (Proquest, Embase, Scopus, Science Direct, PubMed) and for the objective of guaranteeing inclusivity in the literature reviews, we searched Google Scholar and references within the selected articles. The search was performed using a combination of key terms, including reform, policy, intervention, PHC, primary healthcare or primary health care (complete search strategy show in Appendix S2).

### Study selection and data extraction

**Figure 1.**
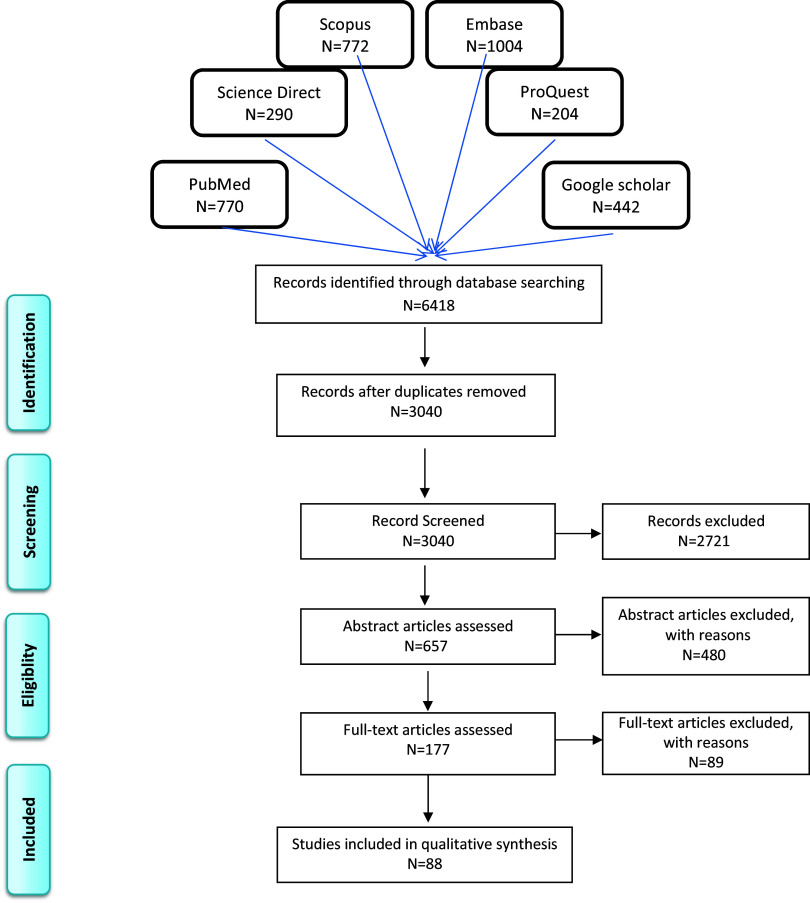
Diagram of the selection of articles for review.

Results from the bibliographic databases were merged, and duplicates were removed. Articles were included if they had relevant information about reforms in PHC. Two researchers (LD and ASh) independently screened and reviewed the titles, abstracts, and full text. Then discussed the findings, and disagreements were resolved by discussion and consensus. Figure 1 shows the number of papers included and excluded at each phase of the selection process. Data on PHC reforms were extracted and entered in the assessment form. And at this stage too, disagreements were resolved by discussion and consensus.

### Synthesis of results

The extracted data were analyzed using the thematic framework approach (Smith and Firth 2011). The study results were further categorized using the WHO’s six building blocks of the Health System Framework, including service delivery, health workforce, information, medical products, vaccines and technologies, financing, and leadership/governance (Hsiao and Burgess 2009).

## Results

### Selection and Characteristics of sources of evidence

Of the 3482 articles identified, 88 studies met the eligibility criteria. No additional studies were identified through manual search. The main reason for exclusion was not addressing reforms regarding primary health care. The studies described PHC reforms in 41 countries (more detail is shown in Table 1), including New Zealand (Gauld 2001, Tenbensel 2008), China (Xu *et al.*, 2007, Tang, Meng et al. 2008, Yip and Hsiao 2009, Hu *et al.*, 2011, Sun *et al.*, 2014, Lin *et al.*, 2015, Di Liang *et al.*, 2020, Pu, Huang et al. 2020, Ran *et al.*, 2020, Tao *et al.*, 2020, Zhou *et al.*, 2020), Chile (Bastías, Pantoja et al. 2008, Unger, De Paepe et al. 2008, Cornejo-Ovalle *et al.*, 2015), South Africa (Benatar 2004, De Maeseneer and Flinkenflögel 2010, Van Pletzen *et al.*, 2013, Schneider, English et al. 2014), Malaysia (Yu, Whynes et al. 2011), India (Ghosh 2014, Rahman, Angeline et al. 2014), Mexico (Frenk, González-Pier et al. 2006, van Weel, Turnbull et al. 2016), United Arab Emirates (Koornneef, Robben et al. 2012, Koornneef, Robben et al. 2017), Georgia (Gamkrelide *et al.*, 2002, Gotsadze *et al.*, 2005), USA (Blumenthal and Dixon 2012, Lankarani 2012, Harrill and Melon 2021, O’Mahen and Petersen 2021), Turkey (Tatar and Kanavos 2006, Yasar 2011, Hone, Gurol-Urganci et al. 2017), Philippines (Obermann, Jowett et al. 2008), Finland (Tynkkynen *et al.*, 2016), Spain (Larizgoitia and Starfield 1997), Kosovo (Buwa and Vuori 2006, Percival and Sondorp 2010), Sweden (Spak and Andersson 2008, Forsberg 2018, Mosquera *et al.*, 2021), Poland (Mokrzycka, Kowalska-Bobko et al. 2016), Australia (Baum, Freeman et al. 2013, Baum *et al.*, 2016), Denmark (Setlhare 2016), Ethiopia (Bradley *et al.*, 2012), Armenia (Grigoryan 2005), Commonwealth of Independent States of Central Asia (Parfitt 2009), Slovenia (Vab 1995), Uganda (Tashobya 2004), Albania (Hotchkiss, Piccinino et al. 2005), South America (Ramírez *et al.*, 2011, Acosta Ramírez *et al.*, 2016), Brazil (Almeida *et al.*, 2000, Kuchenbecker and Polanczyk 2012, Soranz *et al.*, 2016, de M Pontes and Santos 2020), Bosnia and Herzegovina (Atun, Kyratsis et al. 2007), Kazakhstan (Organization and UNICEF 1997, Abzalova *et al.*, 1998), Croatia (Harvey, Kalanj et al. 2004), Portugal (Szczygieł *et al.*, 2011, Biscaia and Heleno 2017), Canada (Harris, Green et al. 2015), Cuba (Sixto 2002, Whiteford and Branch 2008), Lithuania (Liseckienė 2009, Buivydiene, Starkiene et al. 2010), Greece (Tragakes and Polyzos 1998, Tountas, Karnaki et al. 2002, Myloneros and Sakellariou 2021), UK (Blumenthal and Dixon 2012), Ecuador (Quizhpe *et al.*, 2020, Jimenez and San Sebastián 2021), Romania (Bara *et al.*, 2002), Cypriot (Pallari *et al.*, 2020), and Iran (Shadpour 2006, Lankarani 2012, Esmailzadeh *et al.*, 2013, Lankarani *et al.*, 2013, Asaei 2014, Malekafzali 2014, Heshmati and Joulaei 2016).

**Table 1.** Study characteristics

First author	Country/Date	Study Goals	Study Design	Data Collection Method	Formulation and Implementation process	Challenges faced during implementation
Gauld R (Gauld 2001)	New Zealand (2001)	Providing a detailed account of several waves of structural change at the health system level.	Perspective	Original data	NA	NA
Tenbensel T. (Tenbensel 2008)	Canada and New Zealand/2008	Comparing the ways in which governments in Canada and New Zealand have attempted to pursue reforms in two major health policy arenas – cost control and primary health care – in the period 1992–2005.	Qualitative	Review of report	NA	-In New Zealand, the preferences of right and left parties limited forming PHC based on market forces or moving towards community values. -In Canada, the power of medical professionals lead to the predominance of state-profession corporatism in governance of PHC reforms.
Hu R (Hu *et al.*, 2011)	China/2011	Examining health care reform in urban and rural China	Perspective	Original data	-Fiscal decentralizing and introducing market competition and marketization of hospitals. -Shifting a part of the health care financing burden to patients. -Consolidation of health insurance schemes. -Liberation of Medicine prices.	-Reducing the ability of central government to allocate resource to subsidize the poor region. -Unnecessary health provision and health expenditures. -Increasing inefficiency in health system.
Lin F. (Lin *et al.*, 2015)	China/2015	Examining the successful development and innovation experience of its primary health care service system during the new health reform in China since 2009.	Qualitative	Review of documents and regulations, fieldwork, and expert discussions	Primary care system in Hangzhou was implemented through following reforms: -Using government section and unified information platform; -General practitioners as health gatekeepers; -Integration of urban and rural health services and insurance coverage; and -Integration of health care–pension–nursing and using GP-contracted smart services.	NA
Sun C. (Sun *et al.*, 2014)	China/2014	Examining the challenges Facing the Chinese Health Care System	Perspective	Original data	NA	NA
Tang S. (Tang, Meng et al. 2008)	China/2008	Examining challenges to health equity in China	Perspective	Original data	NA	-Combination of rapid economic growth and commercialization which led to notable market failures. -Inequitable distribution of social determinants of health
Xu L. (Xu *et al.*, 2007)	China/2007	Examining urban health insurance reform and coverage in China	Quantitative	Collecting original data	NA	-Population coverage was not achieved. -The diversity in insurance systems continued and deepened. -Partial consolidation of two main governmental schemes. -Rapid economic expansion in urban areas led to massive rural to urban movement which in turn caused an increase in urban population to be covered by health insurance. -There was no governmental subsidy for the self-employed.
Yip W. (Yip and Hsiao 2009)	China/2009	Examining the reform is likely to achieve its stated goal of assuring every citizen equal access to affordable basic health care	Perspective	Original data	The Chinese government declared in April 2009 that it will increase financial resources to invest in five specific areas: -(1) expand insurance coverage with emphasize on rural population and urban uninsured; -(2) increase government spending on public health services, especially in lower-income regions, with the goal of equalizing public health spending across regions; -(3) establish primary-care facilities—community health centres in urban areas and township health centres in rural areas as gate-keepers; -(4) reform the pharmaceutical market; and -(5) pilot test public hospital reforms.	-The reform failed to address the root causes of the wastes and inefficiencies such as a fragmented delivery system and provider incentives to over-provide expensive tests and services. - Moving from fee-for-service payment to DRG or capitation with pay-for-performance was suggested to overcome inefficiencies.
Tao W (Tao *et al.*, 2020)	China/2020	Describing the social, economic and health context in China, and then reviews the overall progress of healthcare reform	Perspective	Original data	Following steps were proposed to achieve universal health coverage: - Constant political support - Increasing health financing -Encouraging investment from both government and private sectors -A strong PHC system should be regarded as a core component in realizing UHC.	NA
Zhou S (Zhou *et al.*, 2020)	China/2020	Aiming to evaluate the impact of the Beijing Reform on healthcare-seeking behaviour	Quantitative	Monitored and statistical data of 373 healthcare institutions	NA	NA
Ran Y. (Ran *et al.*, 2020)	China/2020	Aiming to evaluate the actual effect of China’s Medical Alliances reform in rural areas	Quantitative	Collecting original data	NA	NA
Pu X (Pu, Huang et al. 2020)	China/2020	Aiming to evaluate a pre/post-reform pilot study from 2015 to 2018 in a rural county. The Shengzhou payment reform aimed to shift the passive budgeting payments toward strategic purchasing.	Quantitative	Collecting original data	- At the facility level, a mixed system of input-based (line-item budget) and categorized output-based payments was launched - At the individual level, a basic salary plus a bonus based on performance was given to incentivize Primary Care Practitioners.	- There was no strong data systems for collection, measurement, and reimbursement calculations. - For indicators that could not be included in the IT system during the project, an auxiliary data entry interface was provided as an interim measure.
Di Liang LM. (Di Liang *et al.*, 2020)	China/2020	Building a People-Centred Integrated Care Model in Urban China	Qualitative	Semi-structured interviews and focus groups	-System integration (Integrated primary care and specialty care, Integrated health care with public health services And social services), -Organizational integration (Merged the resources of five district-level public hospitals and 23 public community health centres and founded single legal personhood), -Professional integration (Encouraged specialists to work part-time at community health centres), -Clinical integration (a formal two-way referral system was established, Provided primary care doctors with timely decision support from specialists at hospitals)	-Lack of qualified providers to strengthen primary care. -Primary care facilities often lacked the capacity to provide high-quality health care - Local residents did not trust health centres.
Bastías G. (Bastías, Pantoja et al. 2008)	Chile/2008	Examining health care reform in Chile	Perspective	Original data	-A list of 56 health conditions and treatments was developed to be covered by law. -To develop above list, following criteria were used: burden of disease, effectiveness of treatments, capacity of the health system, financial burden, and social consensus.	-There was a strong distrust by many health professional associations in the performance of the health system reform. -Lack of transparency in the initial priority-setting process -Unsolved problems with the central information system where data were manually codified. -Many health professionals did not recommend the use of guarantees, even though they were obliged to do so by law.
Cornejo-Ovalle M. (Cornejo-Ovalle *et al.*, 2015	Chile/2015	To assess the impact of P4P on the efficiency of primary oral health care providers in Chile.	Quantitative	Secondary research data	-Additional money was provided for Primary Health Care (PHC) teams by implementing Pay for Performance (P4P), - P4P indicators were seniority and training, and collective compliance of performance indicators. -Indicators were defined under technical criteria and negotiated in tripartite committees composed of representatives of PHC Municipal Entities administrators, unions representing the workers and the Ministry of Health. -It was recommended that P4P implementation should apply other quality performance indicators.	NA
Unger J-P. (Unger, De Paepe et al. 2008)	Chile/2008	Assessment and Critique Chile’s Neoliberal Health Reform			NA	NA
Benatar SR. (Benatar 2004)	South Africa/2004	Examining South Africa’s health policies over the past 10 years.	Qualitative	Review of report	-Construction of hundreds of new clinics that provide primary health care, -Integration of medical services, -Making health services free to expectant mothers and children under five years of age, -Development of new food programmes for 5 million children. - Redistribution of resources from tertiary care toward long-neglected primary and community-based care.	-Many new clinics and the district health system were suffering from lack of personnel and finances, poor administration, and expanding demands. -In the public sector, medical jobs were being shifted to new tertiary care institutions in previously disadvantaged areas, but it was difficult to recruit staff to work in such regions.
De Maeseneer J. (De Maeseneer and Flinkenflögel 2010)	Africa/2010	Examining primary health care in Africa	Qualitative	Review of report	-Postgraduate family medicine training for local doctors was developed within the faculty of medicine. -The trainees were working in training sites, mainly in district hospitals, under supervision of family physicians, with input of other specialists when needed.	-Vertical disease oriented programmes, funded by international donors, ‘extract’ the skilled local health personnel out of the local primary healthcare system which had devastating consequences on family medicine.
Schneider H. (Schneider, English et al. 2014)	South Africa/2014	Describing the catalysts to implementation of a whole-system intervention	Mixed Method	Interview, observation, secondary data	-“Primary health care (PHC) outreach teams” were introduced to revitalize the primary health care system. -A team of generalist community health workers (CHWs), supported by a professional nurse, worked in the PHC outreach team. -This team was responsible for a defined number of households and will form close links with the local health facility. - These teams were to provide a wide range of services including HIV/TB, maternal-child health and chronic non-communicable diseases, based on preventive/promotive focus and using intersectoral action and the social determinants of health approach. - A set of programmes including the roles and composition of the PHC Outreach Teams, training materials, a new monitoring and evaluation system integrated into the district health information system were defined. -A partnership with a national non-governmental organization was formed to secure technical support for planning. This partnership played a major role in supporting the implementation process: collecting and providing relevant information, designing the implementation strategy and engaging with front line providers. - A number of structures to support implementation were established including the PHC Re-engineering Task Team, secondment of a full time coordinator or “champion” from the provincial structures, and the appointment of coordinators at district and sub-district levels.	-Absence of dedicated financial resources from national or provincial government. -Primary health care clinic managers who were not well informed and did not fully own the strategy; -Team leaders faced with higher workload as they were appointed from existing staff establishments -Limited number of professional staff to lead teams.
Van Pletzen E. (Van Pletzen *et al.*, 2013)	South Africa/2013	Assessing the size, characteristics and partnership networks of health-related non-profit organizations	Quantitative	Original data	NA	NA
Yu CP. (Yu, Whynes et al. 2011)	Malaysia/2011	Assessing the potential equity impact of Malaysia’s projected reform of its current tax financed system towards National Health Insurance (NHI)	Quantitative	Original data	- NHI promotes the idea of shared responsibility for healthcare among citizens. - Wealthier individuals are expected to subsidize healthcare costs for the less privileged. - Financial burden of healthcare services will be distributed based on individual’s ATP. - Government will allocate funds from MOH’s budget to support the poor and vulnerable. - Government contributions will also help subsidize healthcare for employees and pensioners. - NHI contributions will be collected from income-generating individuals. - Contributions are mandatory for those who can afford to pay, regardless of dependents or services consumed. - Proposed NHI contribution rate is 5% of salary for employees and income for self-employed.	NA
Ghosh S. (Ghosh 2014)	India/2014	Examining health sector reforms and changes in prevalence of untreated morbidity, choice of healthcare providers among the poor and rural population in India	Quantitative	Original data	- User fees were introduced in the public health sector during the eighth five-year plan. - World Bank-sponsored health systems development projects in the late 1990s to early 2000s increased user fees in public hospitals. - Decentralization of healthcare system in the 1990s transferred authority from central government to local levels. - Local governments now have more control over resource allocation and service delivery. - Public spending on health declined during economic liberalization. - Introduction of Drug Price Control Order in 1995 led to liberalization of pharmaceutical sector in 2002.	
Rahman SM. (Rahman, Angeline et al. 2014)	India/2014	Examining role of family medicine education	Perspective	Original data	To achieve UHC, following steps were emphasized on: -Improving the availability of human resources in delivering primary care. -Increasing the availability of trained primary care health workers (doctors, nurses, and mid–wives) to achieve WHO norms -Training rural health practitioners and compulsory rural service for medical graduates after medical school. -Introducing distance learning certification in managing common problems. - Equipping manpower at PHCs and community health centres (CHCs) with skills necessary to meet the common health care needs of the local community.	-Continuing trend toward specialization among medical graduates and fragmented health provision generated varying results. - Ambulatory services for episodic illnesses in the community remained the most common platform for providing patient care. - Focus of under–graduate medical education remained predominant in the teaching hospitals. - Mismatch of medical professional education to patient and population needs, minimal contextual understanding, episodic encounters and predominant hospital orientation at the expense of primary care. - Faculty development, curricular content and goals, location of training and the type of assessment should be changed according to “the family medicine attitude” principles
Frenk J. (Frenk, González-Pier et al. 2006)	Mexico/2006	Providing evidence and background information for the analytical and empirical	Perspective	Original data	- Separation of health-related public goods funding from personal health services aiming to shield public-health activities from being neglected	- Requiring additional public funding to reduce out-of-pocket expenditure and to meet the costly demands associated with the epidemiological transition, especially for complex hospital-based interventions.
van Weel C. (van Weel, Turnbull et al. 2016)	Mexico/2016	Developing an action plan and build leadership capacity	Qualitative	Interview	NA	NA
Koornneef EJ. (Koornneef, Robben et al. 2017)	United Arab Emirates/ 2017	Reviewing the progress and outcomes of health systems reform in the UAE.	Systematic review	Review of articles	NA	NA
Koornneef EJ. (Koornneef, Robben et al. 2012)	United Arab Emirates/ 2012	Describing and review the health system reform in Abu Dhabi to date	Perspective	Original data	NA	NA
Gamkrelide A. (Gamkrelide *et al.*, 2002)	Georgia/2002	Providing detailed descriptions of health systems in the countries of the WHO European Region	Perspective	Original data	- The Government of Georgia defined an essential package of services, or basic benefit package (BBP), funded through central and local budgets and mandatory health insurance contributions. - The BBP aimed to reorient the health system towards public health and primary care from expensive and inefficient secondary care services. - To maintain access to primary healthcare (PHC) services for rural residents: - In 2001, the Government of Georgia implemented the ‘Rural Health Program’ (RHP), partially reversing decentralization in primary care financing. - The RHP provided funding from the Social Insurance State United Fund to contracted PHC providers outside of major cities. - Capitation-based payments were used for PHC teams serving 1500-2000 people in catchment areas. - Local municipalities were responsible for reimbursing maintenance costs of primary health facilities. - The RHP ensured steady funding for rural providers and stable incomes for PHC personnel during 2001-2002.	- Imbalance between state liabilities and resources deepened after additional services were included in the BBP without corresponding budget increases. - Adequate public financing for the sector was lacking, impacting reform implementation due to poor fiscal performance. - Rural populations faced difficulties as healthcare professionals moved to urban areas due to low rural economic status and inadequate public financing. - Many rural clinics and nursing posts closed or struggled to provide basic services due to lack of maintenance, medical equipment, and pharmaceuticals.
Gotsadze G (Gotsadze *et al.*, 2005)	Georgia/2005	Aiming to contribute to the assessment of the impact of health sector reforms	Quantitative	Household survey	NA	NA
Blumenthal D. (Blumenthal and Dixon 2012)	USA and England/ 2012	Identifying three areas relating to financing, organization, and information technology	Perspective	Original data	NA	NA
Lankarani KB. (Lankarani 2012)	USA/2012	Addressing Health Disparities in Mississippi Delta based on Iran’s Health House Model	Perspective	Original data	NA	NA
Harrill WC. (Harrill and Melon 2021)	USA/2021	Attempting to clarify working definitions and conceptual boundaries within the lexicon of U.S. healthcare reform	Review	Pubmed/MEDLINE/Google search.	The Patient-Centered Care Model (PCCM) was implemented using 8 strategies: -(a) respect for the patient’s preferences, -(b) coordination and integration of care, -(c) information and education, -(d) physical comfort, -(e) emotional support, -(f) involvement of family and friends, -(g) continuity and transition, and -(h) access to care. In Patient-Centered Medical Home (PCMH) model was implemented using following strategies: - (a) physician-stakeholder providing initial contact, continuous and comprehensive care within the physician-patient relationship, -(b) a physician-directed medical care team, -(c) a whole person orientation of care through all stages of the patient’s health cycle from preventative care to acute and chronic care and finally, end of life care, -(d) coordination across all elements of the patient’s care plans, -(e) incorporation of quality and safety metrics within patient reported outcomes, evidence-based medicine, continuous quality improvement, healthcare information technology data and communication, and professional recognition standards, -(f) enhanced patient access strategies for care availability, and -(g) payment reform that recognizes the added value to the patient rather than the volume of services consumed.	
O’Mahen PN. (O’Mahen and Petersen 2021)	USA/2021	Examining how potential reforms influence state-federal relations	Perspective	Original data	NA	NA
Hone T. (Hone, Gurol-Urganci et al. 2017)	Turkey/2017	Introducing family medicine across all 81 provinces of Turkey	Quantitative	Surveys	Key changes to health system functions related to primary health care implemented in Turkey (2003–10). Organization and governance - New contracting arrangements between the Provincial Health directorate and family medicine (FM) staff were established with performance-based capitation payment. -FM units, comprising FM doctors, nurses and midwives, were established to provide integrated diagnostic, therapeutic and preventative care as the first point of service. -Formal referral system to secondary and tertiary care was abolished, but people were encouraged to visit PHC first. -Capitation payment for FM doctors was based on socioeconomic development of region served with a bonus of up to 40% of the base payment in historically underserved areas; -20% of the salary was performance-based dependent on meeting targets including as immunization, antenatal care, and child registration -Pay for performance was expanded in 2006, to include 35 administrative indicators. -Co-payments (for public hospitals and private hospitals) were introduced in 2009 for outpatient and secondary care without referral from primary care. -Cost sharing for PHC services was removed.	NA
Tatar M. (Tatar and Kanavos 2006)	Turkey/2006	Aiming to health care reform in Turkey	Perspective	Original data	- Gatekeeping system was implemented in 1998 in France. - Under per capita annual payment programme, these doctors accepted to provide public health services, to keep patients’ medical records, and to prescribe cheaper drugs.	NA
Yasar GY. (Yasar 2011)	Turkey/2011	Assessing the ‘Health Transformation Programme’ (HTP) in Turkey announced in 2003	Qualitative	Brief history of health policy	-A pilot family medicine was implemented using capitation payment. - Family physician was responsible for the health of all members of a family. - Family physician provide all health services within the scope of the primary health care services and more sophisticated health problems are referred to a specialist or a dentist. -Primary health centres provide integrated preventive, diagnostic, curative and rehabilitative services and are responsible for overseeing preventive health services like vaccination campaigns and reproductive and child health services. - People not following the referral chain will be excluded from insurance and will have to pay their full expenses.	-The ratio of family physicians to population was low in Turkey.
Obermann K. (Obermann, Jowett et al. 2008)	Philippines/2008	Aiming to lessons for health care reform from the less developed world	Perspective	Original data	NA	NA
Tynkkynen L-K. (Tynkkynen *et al.*, 2016)	Finland/2016	Describing the current policy debates and initiatives promoting the expansion of the choice of primary care provider in Finland	Perspective	Legislation and policies	- Residents were previously limited to the primary health care unit based their living place, but after the reform, residents had been allowed to change the unit once a year. - The Government outlined that in the future patients and customers will be able to choose between public, private, or third sector providers. - This reform aimed to tackle the serious flaws in the current service system, especially long waiting times at the level of primary health care, through enhancing patient choice and competition. - At the same time, the multi-source funding system was planned to be abolished.	Challenges: - The under-resourcing of primary health care fitted poorly with the patients seeking care in the municipal primary health centers of Finland. - In municipal primary health care, the majority of the patient visits were made by people who do not have the opportunity to use occupational health care or private services, i.e. children not covered by voluntary private health insurance, the retired, the unemployed, and other people in weaker labour market positions. - People in this group also have relatively greater and multiple care needs and an increased tendency to suffer from chronic diseases. - Lack of a consistent remuneration system and the lack of economic incentives for the providers, hindered the patient choice. - There was a relatively broad variation in remuneration practices between and even within municipalities. - For a health centre unit, it was mostly attractive only to take care of the population it was responsible for and not to attract more patients from other areas of the same municipality because they were paid on a capitation basis. - However, it might be profitable to attract patients from other municipalities because in such cases, the remuneration was usually arranged on a fee-for-service basis instead of capitation. - Primary health centres were suffering from staff shortages and diminishing resources which made it difficult to attract patients from other municipalities.
Larizgoitia I. (Larizgoitia and Starfield 1997)	Spain/1997	Assessing an incremental reform initiated in Spain 10 years ago	Qualitative	Interview	-In new approach, primary care focused on team work as the mode of practice to increase the accessibility, comprehensiveness, coordination of care and patient’s satisfaction, rather than solo practice and episodic care as delivered previously. -Area of influence: Geographically defined population base (the ‘health zone’) -Place of delivery: Health care centres: new physical and functional structures to perform PHC activities. -Work organization: Team work -Staff Multidisciplinary team, formed by family physicians, nurses, midwives, paediatricians, and others under the direction of a medical coordinator -Content of care: Delivery of care at a health care centre or at home, coordinated with other levels of care, prevention, health promotion, education, needs assessment and evaluation of activities. -Attributes of care: Longitudinal, continuous, integrated and comprehensive care, based on team work of all professionals.	NA
Buwa D. (Buwa and Vuori 2006)	Kosovo/2006	Exploring the complexity of a health care system reforms in a post-conflict situation	Qualitative	Interview	-The cornerstone of the policy will be PHC that emphasizes family medicine and acts as gatekeeper to secondary and tertiary care. -The municipal health houses will turn into family health centres with 24-h coverage providing preventive, curative, dental, and emergency services. -Main focus of PHC would be on maternal, child, adolescent, and reproductive health. -Each family doctor provides services for 2000 family members. -People will register with a family physician from the nearest health centre. -Family physicians was supposed to gradually replace the clinical specialists in the family health centres and receive a capitation fee for each patient on their list. -The family medicine team will initially consist of family doctors and nurses. As their numbers increase, other primary care workers, such as public health nurses, midwives, physiotherapists, occupational therapists, social workers, and psychologists will join in. -The team will use common patient records. -The Ministry will promote community-based services for pre- and post-natal care, rehabilitation and mental health and for the treatment of many chronic diseases, e.g. diabetes and hypertension. -Child psychiatry will be developed -The Ministry will promote a handicapped friendly society. -The Ministry was supposed to dismantle vertical programmes and integrate their useful elements either with primary care or with hospitals. -Aiming at dismantling the old, centralized ‘command and control’ management system, a regulation was passed that made the municipalities responsible for PHC, consumer protection, and public health.	-Dismantling of vertical programmes The Ministry transferred the immunization programme from the Institute of Public Health to the municipal PHC and closed many tuberculosis dispensaries. However, the threat of an epidemic seemed to justify a new vertical HIV/AIDS control programme. - The implementation of decentralization of rehabilitation services was not satisfactory. - HIS was not able to provide reliable data on many key indicators needed for policy planning and decision making. - Governmental budget for health diminished. -Hospital doctors find PHC a useless ‘prescribe-and-refer’ revolving door. -PHC doctors are fleeing from family medicine to the clinical specialties. -Working in the public sector was for many young doctors a ‘necessary evil’ until they have enough expertise or money to establish a private practice. -Established clinical specialists, diverted the wealthiest patients from the public sector to their private practices. -The devolution of primary care to the municipalities took place too early. Many municipalities admit that they are not ready and that they have neither the requisite professional nor the managerial capacity. -The flight of doctors to hospital specialties has seriously damaged PHC. -The Ministry was not doing much to retain those already in PHC or to attract others to it. -The Ministry’s preoccupation with ‘high-tech’, made the municipalities to be more interested in clinical specialists than family physicians.
Percival V. (Percival and Sondorp 2010)	Kosovo/2010	Developing a framework for analyzing health reform in post-conflict settings	Review	Relevant literature	-Family medicine teams operating in primary care centres would provide initial diagnoses and curative care, with the objective of treating 80 to 90 percent of presenting problems. -The location of health clinics would be determined on the basis of population. -Facilities would have catchment populations of approximately 10,000 individuals. -Family medicine centres would be responsible for diagnoses and curative care, including minor surgery and drug management; emergency care and stabilization of emergency patients; maternal and child healthcare; and reproductive health services, including antenatal and post-natal care, as well as family planning and treatment of sexually transmitted diseases. -Family doctor are responsible for coordinating specialist and tertiary-care services. -Patients who bypassed the referral system would face a financial penalty. -Prevention activities such as health education and immunization would be run out of these centres, as would services such as home visits, palliative care, community rehabilitation, and community mental health services.	NA
Forsberg E. (Forsberg 2018)	Swedish/2018	Impacting of the Swedish Patient Choice Reform	Qualitative	Collecting data at a regional level	-People were allowed to choose between private and public providers in the primary health care sector on health care utilization aiming to increase freedom of choice and expansion of the primary health care.	NA
Spak F. (Spak and Andersson 2008)	Swedish/2008	Aiming to describe the Swedish efforts to implement secondary prevention of alcohol problems on a large scale in Primary Health Care	Perspective	Original data	NA	NA
Mosquera PA. (Mosquera *et al.*, 2021)	Swedish/2021	Assessing whether the reform have impacted on primary health care service performance	Quantitative	Ecological register-based	-Private primary health care (PHC) providers were introduced. -national Free Choice in PHC (FCPHC) reform allowed all PHC providers with certain basic requirements to establish a health care centre at a geographical location of their choice -It allowed patients to choose their PHC. - Capitation payment was used for the providers. -The responsibility of the PHC centres was transformed from the entire population in a catchment area, to only the patients listed at that specific centre. -The reform aimed to expand the PHC provision and to introduce competition between health care centres, in order to improve efficiency and quality of services.	-There were concerns about shifting from a more egalitarian to a more libertarian view of health care organization. -Other concerns were about reduced continuity, increased fragmentation and impaired equity in the provision of services.
Mokrzycka A. (Mokrzycka, Kowalska-Bobko et al. 2016)	Poland/2016	Exploring legislative changes directly and indirectly affecting primary health care (PHC)	Perspective	Original data	NA	-The number of family medicine physicians in Poland was low (one per 3500 people, compared to one per 2500 recommended by the experts). - Demand for paediatricians was declining due to the aging of the population -The Minister of Health allowed all specialists in internal diseases and paediatrics to work in the statutory health care system as PHC physicians and to set up their own (solo) PHC practices. The policy that came into force in June 2014 included the following measures: -The legal requirement on primary care doctors with less than ten years of experience in PHC prior to the adoption of Directive 2005/36/EC to specialize in family medicine by 2017 was expunged; and -All paediatricians and internists, not only those who had at least ten years of experience in PHC prior to the adoption of Directive 2005/36/EC, were formally allowed to work as PHC doctors.
Baum F. (Baum, Freeman et al. 2013)	Australian/2013	Reporting on the health promotion and disease prevention conducted at Australian multi-disciplinary primary health care	Qualitative	Interviews	NA	NA
Baum F. (Baum *et al.*, 2016)	Australian/2016	Applying a critical analysis of the impact of neo-liberal driven management reform to examine changes in Australian primary health care	Qualitative	Interviews	-Selective PHC, with its ‘vertical’ emphasis on treating or preventing certain high-burden diseases rather than a ‘horizontal’ effort to build public health systems, became more entrenched with health reform initiatives of the 1990s and 2000s that were consistent with the core elements of neo-liberalism: cost-containment and efficiency, result-based financing, user fees, managed competition amongst service providers, increased contracting out to private providers, and an emphasis on individual responsibility for maintaining good health.	NA
Setlhare V. (Setlhare 2016)	Denmark/2016	Experiencing in countries with good health indicators can help inform discussions on the future of family medicine in Africa	Qualitative	Observations	-Task shifting facilitated Danish PHC efficiency. - Danish PHC was supported by a reliable modern Information Technology (IT) system. - FM physicians training in Danish is different and longer than that in Southern Africa. -PHC services are provided free of charge by the Danish government -Danes register with a FP who attends their medical problems and they are free to change FPs at not too frequent intervals. -Approximately 30% of a Danish FP’s salary is guaranteed. The rest of the salary is proportional to the number of patients the FP consults. -Danish FPs act as gatekeepers to the rest of the health system. -The Danish FP has ultimate responsibility for patients in his care and this promotes continuity of care, gate keeping and efficiency in healthcare delivery. -FP training in Denmark includes an initial 6 months of FM residency in a FP-run clinic. This is then followed by 6 months in another FP-run clinic where formal training in FM begins. Subsequently, there are rotations of 6 months each in internal medicine, paediatrics, surgery, obstetrics/gynaecology, and psychiatry. The last leg of training is a 12-month stint in a clinic working almost independently.	NA
Bradley EH. (Bradley *et al.*, 2012)	Ethiopia/2012	Examining the variation in impact of a health systems strengthening intervention	Mixed methods	Longitudinal data	NA	NA
Grigoryan A. (Grigoryan 2005)	Armenia/2005	Describing new model of care and lessons learned	Perspective	Original data	NA	NA
Parfitt B. (Parfitt 2009)	Central Asia CIS countries/2009	Examining the key human resource issues for health amongst mid-level workers	Perspective	Original data	Uzbekistan Following initiatives were developed to strengthen primary health care services: -Educating and retraining of nurses and doctors -Establishment of Training centres for the retraining of specialist doctors as General Practitioners within medical schools and at Family Physician training centres. -Initiation of a programme to develop the expertise of nurses and retrain them as family or generalist community nurses. -Focus of the nurses on maternal and child health care. -Similar programmes have been developed in Kirghizstan, Kazakhstan and Uzbekistan.	-The major challenge in all of these countries was the lack of higher qualified nurses who were eligible to teach.
Vab I. (Vab 1995)	Slovenia/1995	Explaining the main features of the primary health care system and its reform in Slovenia	Perspective	Original data	-The health centres were established in order to provide universal coverage for the population of a given area and to control either important health problems (e.g. tuberculosis) or to take care of groups of people at risk (e.g. pre-school children). -Their role was very community oriented. -Every health centre had to have general practitioners, dentists, a paediatrician, a gynaecologist, a specialist in school medicine, a specialist in industrial medicine, district nurses, physiotherapy and laboratory. -Financing of the health centre was provided through a budget according to the estimated needs of the population for primary care. -The structure of the health centre is not strictly defined. Primary health care can be maintained by different health professionals: the GPs alone or in combination with other health professionals (paediatricians, gynaecologists etc.). -The health centre is not the exclusive provider of primary health services in the community.	
Tashobya CK. (Tashobya 2004)	Uganda/2004	Describing primary health care and health sector reforms in Uganda	Perspective	Original data	-From 1980 to 1983, there was a contention on whether to implement comprehensive PHC or selective PHC took place. -Selective PHC was the preferred strategy. So the introduction of vertical programmes/projects took place defeating the idea of horizontal holistic implementation of PHC programmes. -These are some of the main vertical programmes: The Control of Diarrhoeal Diseases programme, Growth monitoring programme, Oral rehydration therapy, Breast feeding, Immunization, Food security, Family planning, and Female education.	- The preference of vertical programmes led to fragmented and uncoordinated implementation of PHC in Uganda. - Government had no control on the vertical programmes which had substantial and considerable external/donor funding. -There was inefficient use of these scarce public resources, which were largely spent on inappropriate and cost-ineffective; emphasis on tertiary rather than primary care. To address these challenges, following strategies were proposed; -Broadening health financing which included charging users of public facilities, -Providing health insurance or other risk coverage and establishing community pre-payment schemes; -Decentralization of health services; -Privatization and broadening the provider mix with emphasis on effective use of non-governmental resources and targeting improvements in human resource management.
Hotchkiss D. (Hotchkiss, Piccinino et al. 2005)	Albania/2005	Impacting assessment of a pilot project	Perspective	Original data	The PHC pilot intervention composed of following projects: -Purchasing new equipment for pilot facilities; -Training providers in the implementation of clinical practice guidelines; -Revising the medical chart system; -Building management capacity; -Improving community outreach; -Implementing health financing reform; -Constant monitoring the operational processes to evaluate the implementation problems and to measure its impact on availability, utilization, and quality of PHC services.	NA
Ramírez NA. (Ramírez *et al.*, 2011)	South America/2011	Summarizing an extensive review of South American experiences with primary health care since the Declaration of Alma-Ata	Qualitative	Narrative synthesis	NA	-Primary health care was subject to varying interpretations and manifestations (i.e., as an intervention strategy, public policy, level of care and/or model of care). - Unreliable national sources of funding, - Lack of sustainability of programmes tied to external donors or lenders. - Tension between specialist physicians with different backgrounds - Tension between medical and non-medical staff. - Low self-esteem and poor pay among primary health care professionals - Difficulty to persuade physicians to work in poor, rural areas - Tension exists between different conceptual models underlying the health service (i.e., health as holistic and socially determined services vs. biomedical and market determined services), - Tension exists between horizontal and hierarchical approaches to service organization.
Ramírez NA. (Acosta Ramírez *et al.*, 2016)	South America/2016	Aiming to describe and contrast the PHC approaches being implemented in SA to provide knowledge of current conceptions, models and challenges	Qualitative	Interview	-Neo-selective model: In this model coverage is segmented by private and public regimes; here, individual and collective care are separated. -- Comprehensive approach: It is similar to the Alma-Ata model where the public sector predominates and individual, family and community care are coordinated under the responsibility of the same health care team. -In most countries, two main health facility types: posts and centres provide the PHC services. - Health posts serve small, rural, dispersed populations and are generally staffed by nursing auxiliaries or community health workers (CHWs). - Health centre teams commonly comprise a doctor, a nurse, nursing auxiliaries and sometimes a midwife, social assistant, dentist and dental assistant. -In six countries (Argentina, Chile, Guyana, Paraguay, Peru and Uruguay), PHC teams included nurse-midwives, midwives or obstetrics graduates. -In other cases (for example, in Venezuela and in Bogotá, the capital city of Colombia), there are complementary teams comprising other health professionals, such as psychologists, social workers and environmental technicians.	NA
Almeida C. (Almeida *et al.*, 2000)	Brazil/2000	Focusing on the equity in health care services	Perspective	Original data	NA	NA
Kuchenbecker R. (Kuchenbecker and Polanczyk 2012)	Brazil/2012	Aiming to provide an analysis of some of the most recent changes in the development of HTA in Brazil	Perspective	Original data	NA	NA
Soranz D. (Soranz *et al.*, 2016)	Brazil/2016	Describing and analyzing the main components of the Reform in Primary Health Care (RCAPS)	Perspective	Original data	-Family Health Teams was selected as a basis to deal with low coverage of services at the first level of care and less municipal funding among the capitals of the country change. Reform in Primary Health Care was structured using four essential attributes: - (i) access and provision of first-contact services, - (ii) the assumption of longitudinal responsibility by the patient (continuity of the physician-patient relationship across life) independent of the absence or presence of illness, - (iii) the guarantee of holistic care beginning with a consideration of the physical, mental, and social contexts of health within the limits of the activity of the health teams, and - (iv) the coordination of different actions and necessary services to resolve less frequent and more complex needs. Reform in Primary Health Care was based on three attributes: -(i) the family orientation, -(ii) the community orientation, via epidemiological knowledge of a given locale, -(iii) cultural competency, which refers to the relation between the health professionals with specific cultural characteristics.	NA
de M Pontes AL. (de M Pontes and Santos 2020)	Brazil/2020	Using a historical perspective to contextualize the creation of the Indigenous Health Subsystem in Brazil	Qualitative	Interview	The Indigenous Health Subsystem was implemented in Brazil through following three points: -(1) the centrality of a holistic health perspective; - (2) the emphasis on social participation; - (3) the need for the reorganization of health care.	NA
Atun RA. (Atun, Kyratsis et al. 2007)	Bosnia and Herzegovina/2007	Examining the introduction and diffusion of family-medicine-centred PHC reforms in Bosnia and Herzegovina	Mixed Method	Interview	NA	To further scale up and sustain the reforms, following challenges needed to be addressed: -Developing human resources for the family medicine team, -Expanding services delivered by the family medicine team, -Creating a robust referral and counter-referral system, -Establishing monitoring and evaluation systems, -Harmonizing various approaches to care delivery adopted by different cantons, and -Addressing inequities due to different income levels from health insurance revenues in different cantons.
WHO. (Organization and UNICEF 1997)	Kazakhstan/1997	Describing primary health care and health sector reform	Perspective	Original data	NA	Following problems made the implementation of Alma-Ata declaration difficult in most countries at the beginning: -The distraction of the East-West conflict. - The diversion of funds from development to the arms race. - The debt crisis and poor governance in so many countries. - The inadequacy and skewed nature of foreign assistance. - The final crisis and collapse of the former socialist states. - The shocking increase in poverty in a number of industrialized. - Democracies…and the proliferation of intractable civil and ethnic strife. - Although there was broad agreement on the objective of the Alma-Ata Strategy, it was not clear how to build such comprehensive systems. - Building a comprehensive PHC system was beyond the means of the vast majority of developing countries and even of a range of industrialized countries. - There was a hot debate about different approaches to implement PHC including horizontal vs. vertical programmes, selective vs. comprehensive primary health care.
Abzalova R. (Abzalova, Wickham et al. 1998)	Kazakhstan/1998	Describing reform of primary health care in Kazakhstan and the effects on primary health care worker motivation	Perspective	Original data	-Creation of independent family group practices - Using capitation payment directly financed from the Ministry of Health, - Encouraging free choice of primary care providers through open enrolment, - Creating a non-governmental primary care physician association. - Providing stronger financial incentives for performance, - Ensuring strong feedback mechanisms from the community to care providers, - Engendering a stronger sense of professionalism among primary care providers.	NA
Harvey K. (Harvey, Kalanj et al. 2004)	Croatian/2004	Describing and addressing demand side issues and provided advice aimed at evaluating and improving the quality and effectiveness of drug	Mixed Method	Focus group, sorting CIHI prescription data	NA	NA
Biscaia AR. (Biscaia and Heleno 2017)	Portugal/2017	Aiming to characterize the 2005 reform of Portuguese CSP with an analysis of its systemic and local realms	Qualitative	Documentary analysis and description	-Units called Family Health Units (USF) was established which composed of voluntary and self-organized multidisciplinary teams that provide customized medical and nursing care to a group of people. - The remaining realms of primary health care were reorganized with the establishment of Health Center Clusters (ACeS). - Clinical governance was implemented to improve quality and participation and accountability of all. - The Council of Ministers was established “to conduct the global project of launching, implementing, coordinating and following-up the strategy for the reconfiguration of health centres and implementation of family health units”. - Another strategy was “adaptive policies” which were based on integrated and forward-looking situation analyzes. These policies aimed to ease implementation of Family Health Units through following steps: -Reflection on inputs from as many stakeholders as possible, - Promoting and encouraging varied responses (not just one), - Automatic policy adjustments through the monitoring of key, indicators and lifelong learning, - Focusing on self-organization, social networking and decentralization of governance. To achieve optimization and sustainability of the National Health Service, the Health Centers were reorganized through following principles: -(i) community orientation; - (ii) organizational and management flexibility; - (iii) debureaucratization; - (iv) teamwork; - (v) autonomy and accountability; - (vi) continuous quality improvement; - (vii) contractualisation and evaluation. The process of moving to the “new health centers” also included: -(i) establishment of Family Health Units (USF); - (ii) association of Health Centers in Health Center Clusters (ACeS); - (iii) creation of other functional units in the ACeS - (iv) introduction of a new management model; - (v) implementation of clinical governance; - (vi) reorganization of support services; - (vii) complete computerization of services and dematerialization of most of the support for the practice	NA
Szczygieł N. (Szczygieł *et al.*, 2011)	Portugal/2011	Aiming to present the advances in a recent reform chosen by the policymakers	Perspective	Original data	NA	NA
Harris SB. (Harris, Green et al. 2015)	Canada/2015	Describing the evaluation of the Quality Improvement and Innovation Partnership Learning Collaborative	Mixed method	Survey, interview	Family Health Teams (FHTs) was introduced in 2005 in Ontario, Canada through following characteristics: -(1) group practice and practice networks; - (2) patient enrolment and roistering; - (3) changes to PHC governance and accountability; - (4) funding and compensation; - (5) creation of multidisciplinary care teams including family physicians, nurse practitioners/registered nurses, and other health care professionals (for example, social workers and dieticians); - (6) internet technology infrastructure; and - (7) education/training with a focus on quality improvement.	NA
Whiteford LM. (Whiteford and Branch 2008)	Cuba/2008	Exploring the successes of Cuba’s preventive primary health care system and its contribution to global health	Perspective	Original data	NA	NA
Sixto FE. (Sixto 2002)	Cuba/2002	Evaluation of four decades of Cuban healthcare	Perspective	Original data	-In contrast with other socialist countries focusing on industrialized urban labour force, he Cuba initially concentrated on providing health care to the rural sector. -On January 23, 1960, the Rural Health Service was established which “required all medical school graduates to serve for one year in the rural areas upon graduation and provided for the creation of rural health facilities.	NA
Liseckienė I. (Liseckienė 2009)	Lithuania/2009	Aiming to evaluate changes in family physicians’ task profiles of PHC reform in Lithuania	Quantitative	Combines data from patients and their family physicians	NA	NA
Tragakes E. (Tragakes and Polyzos 1998)	Greece/1998	Tracing out reform plans since the early 1950s	Perspective	Original data	-In 1983, special emphasis was placed on the development of primary health care; - Referral system was established; - A network of urban and rural health centres was established to provide comprehensive primary care services and implementing health promotion and disease prevention programmes; - In January 1994, it was decided to establish a family doctor system, beginning with urban areas. -General practitioners would work in groups or solo practice with lists of about 1500 registered residents. - There was to be free choice of GP. - Existing urban polyclinics and rural health centres, as well as ambulance centres were to be upgraded. - Referral to higher levels of care will be part of the system, but will not be compulsory (hence there is to be no gatekeeping role of primary care physicians). - A new payment methods consisting of fees per registered patient for specific services and capitation were to be initiated in the PHC networks.	NA
Tountas Y. (Tountas, Karnaki et al. 2002)	Greece/2002	Describing reform in the Greek national health system	Perspective	Original data	-Primary Health System was the core of the 200 reform measures announced by the new Minister of Health and Welfare in 2000. - The organization of a Primary Health System in urban areas, and the strengthening of Public Health and Health Promotion. -Creation of urban health centres and the establishment of a personal (family) doctor system. - Personal doctors will be paid based on a combination of three modes of payments. A monthly payment, an annual capitation fee for each person in their list and with bonus of productivity while a fee-for-service system will be the mode of payment for their private practice. - People were to choose a ‘point of reference’ between the nearest urban or rural health centre and a specific personal doctor selected from a list created for each municipality. - The health centre or the personal doctor will have the responsibility of referring patients to hospitals, to special treatment, to clinical laboratory tests etc. - They were supposed to keep an electronic database with the medical records of all their patients.	NA
Myloneros T. (Myloneros and Sakellariou 2021)	Greece/2021	Examining whether the Primary Health Care reforms during that period assisted the country in moving towards Universal Health Coverage	Scoping review	Review of the literature and grey literature	NA	NA
Jimenez SEF. (Jimenez and San Sebastián 2021)	Ecuador/2021	Aiming to assess the impact of the 2008 health reform on the performance of primary health care services in Ecuador	Quantitative	Crude and adjusted rates	NA	NA
Quizhpe E. (Quizhpe *et al.*, 2020)	Ecuador/2020	Assessing whether the health care reforms implemented in the decade between 2007 and 2017 have contributed to reducing the socioeconomic inequalities in women’s health care access	Quantitative	Survey	NA	NA
Bara A-C (Bara *et al.*, 2002)	Romania/2002	Describing health care reforms and analyze the transition of the health care system in Romania in the 1989- 2001 period	Qualitative	policy documents, political intentions and objectives of health care reform	NA	NA
Pallari E. (Pallari *et al.*, 2020)	Cypriot/2020	Assessing the existing state of training, support, quality, guidelines and infrastructure towards a better healthcare system in Cyprus	Mixed method	statistical data and workshop discussions	NA	NA
Lankarani KB. (Lankarani *et al.*, 2013)	Iran/2013	Describing characteristics, progresses and challenges of the Iranian health system	Perspective	Viewpoint of ex-managers	-Iran introduced its own model to implement the principles of Alma Ata. - Health workers called Behvarz were selected from rural inhabitants with elementary education to provide primary health services in rural areas. - They passed the special training programme for 2 years and then appointed as Behvarz to the health house covering up to 1500 people. - Mental health integration in primary care was started to screen for major psychiatric disorders and their referral cases.	NA
Shadpour K. (Shadpour 2006)	Iran/2006	Describing health sector reform in Islamic Republic of Iran	Qualitative	Expert opinion	NA	NA
Esmailzadeh H. (Esmailzadeh *et al.*, 2013)	Iran/2013	Describing Iran Health System Reform Plan Methodology	Descriptive	Plan of Health System Reform	NA	NA
Heshmati B. (Heshmati and Joulaei 2016)	Iran/2016	Describing Iran’s health-care system in the past three decades	Descriptive		-The Family Physician Programme was established in 2005 and implemented in rural areas and cities with populations of less than 20 000 people.	Challenges: -Improper implementation of gatekeeping system - No control on self-refer of patients to specialists. - Absence of financial support, - Fragmentation of insurance system, - Deficiencies in family physician training, and - Conflict of interests.
Doshmangir L. (Doshmangir, Moshiri et al. 2019)	Iran/2019	Aiming to analyze the HTP at the PHC level in Iran	Qualitative	Interview	As a part of “Health Transformation Plan” (HTP) in May 2014, primary health section was improved through following initiatives: - Developing family practice, service delivery and PHC services in rural areas and cities with a population of under 20,000 and in suburban areas and cities with a population of about 20,000 to 50,000. - Integration of new services including smoking cessation, improving nutrition, preventing traffic accidents, promoting physical activity, preventing cancers, cardiovascular disease, and diabetes, improving oral health and preventing mental illness and improving the health status of people with mental illnesses, - Establishing and strengthening intersectoral collaboration, and - Establishments and modification of FP and referral system. - Review of training syllabus for health staff, - Developments of new academic disciplines and on the-job training, - Designing and implementing graduate courses including a Master of Family Medicine (an online modular course) and a family medicine specialty programme, - Developing an online electronic information system; - Establishment of a monitoring and evaluation system for services, - Extensive assessment of clients’ satisfaction.	
Malekafzali H. (Malekafzali 2014)	Iran/2014	Describing to 40 years of Iranian experience in primary health care in west Azarbaijan and behvarzs	Descriptive		NA	NA
Asaei S. (Asaei 2014)	Iran/2014	Describing to Iran’s primary health care system	Perspective	Original data	NA	NA
Saltman RB. (Saltman and Figueras 1998)	Western European/1998	Reporting the results of a broad survey of health care systems in western Europe	Review	Report	NA	NA
Auener S. (Auener *et al.*, 2020)	2020	Focusing on lessons from COVID-19 to increase the sustainability of health systems	Descriptive		NA	NA
Doshmangir L. (Doshmangir *et al.*, 2020)	Iran/2020	Exploring to historically primary healthcare (PHC) development in Iran in the light of development plans	Review	Relevant published and unpublished policy documents	-Launching the Maternal and Child Health Program in 1939 with establishment of the Midwifery School in Tehran (capital city of Iran). - The first nursing school was founded in Mashhad in 1940 to prepare specialized medical teams to serve in villages and rural areas. - The Maternal and Child Health Organization was established in 1940 to support disadvantaged pregnant women by providing outpatient prenatal care. - In April 1964, the Health Corps Law was passed by the parliament, and the first teams of health professionals were sent to rural areas in the same year. - The Health Corps teams consisted of medical university graduates who served their military service as health care providers and some high school graduates who were assigned to health houses in villages after undergoing special training.	NA
Doshmangir L. (Doshmangir *et al.*, 2019)	Iran/2019		Analytical description	Health policy reforms	- PHC network was formed in Iran in 1985. - It aimed to provide public health services to rural and remote areas by non-physician local health workers. - To do so local community health workers, so-called “*Behvarz*” was introduced. - The main focus of PHC network was mother and child care plus tackling communicable disease, which were the main causes of disability and death at that time. - *Behvarz* Training Centers were established in all provinces, whose aims were to train and empower health workers to address the populations’ needs at the community levels.	-Despite being successful in rural areas, the PHC network did not succeed in accommodating the FP programme and the referral system in urban settings. - PHC was not competent to address the emerging burden of NCDs and other evolving social problems.

**Figure 2.**
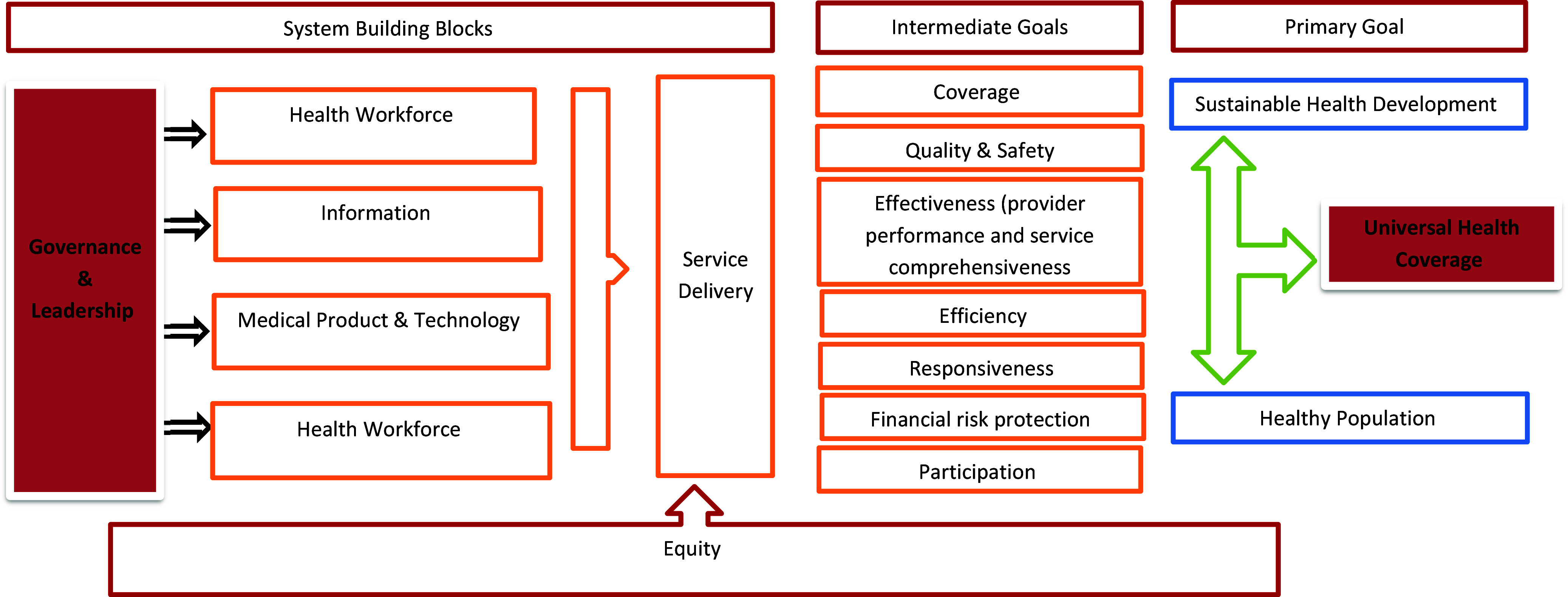
Conceptual presentation of findings.

Table 2 presents a summary of PHC reforms areas based on the six building blocks of the WHO Health System Framework. Only in several countries reforms focused on all six dimensions of the Health System Framework (e.g., Albania (Hotchkiss, Piccinino et al. 2005), Brazil (Almeida *et al.*, 2000), China (Yip and Hsiao 2009), Iran (Shadpour 2006), Portugal (Biscaia and Heleno 2017), and Turkey (Tatar and Kanavos 2006)). In contrast, others prioritized reforms implementation only in one of the Health System Framework blocks (e.g., (Almeida *et al.*, 2000, Sixto 2002, Harvey, Kalanj et al. 2004, Hotchkiss, Piccinino et al. 2005, Hu *et al.*, 2011, Yu, Whynes et al. 2011, Esmailzadeh *et al.*, 2013, Mokrzycka, Kowalska-Bobko et al. 2016, Biscaia and Heleno 2017, Hone, Gurol-Urganci et al. 2017). We further summarize our findings on interventions using Health System Framework building blocks as subsections and Table 2 (detailed overview in Appendix Tables S3, S4, S5). The conceptual presentation of the results can be found in Figure 2.

**Table 2.** Primary health care reforms under the six building blocks

Country	Service delivery	Financing	Health workforce	Leadership/ governance	Medical products, vaccines & technologies	Information
Albania (Hotchkiss, Piccinino et al. 2005)	*	*	*	*	*	*
America (Lankarani 2012)	*	*	*	*		*
Armenia (Grigoryan 2005)	*	*	*	*		*
Australia (Baum, Freeman et al. 2013, Baum, Freeman *et al.* 2016)	*	*	*	*		*
Bosnia and Herzegovina (Atun, Kyratsis et al. 2007)	*	*	*	*	*	
Brazil (Almeida *et al.*, 2000, Kuchenbecker and Polanczyk 2012, Soranz *et al.*, 2016)	*	*	*	*	*	*
Canada (Tenbensel 2008, Oandasan, Conn *et al.* 2009, Strumpf, Levesque *et al.* 2012, Harris, Green et al. 2015)	*	*	*	*		*
Chile (Bastías, Pantoja et al. 2008, Unger, De Paepe et al. 2008, Cornejo-Ovalle *et al.*, 2015)	*	*		*		
China (Xu *et al.*, 2007, Tang, Meng et al. 2008, Yip and Hsiao 2009, Hu *et al.*, 2011, Sun *et al.*, 2014, Lin *et al.*, 2015, Di Liang *et al.*, 2020, Tao *et al.*, 2020)	*	*	*	*	*	*
Commonwealth of Central Asian countries (Parfitt 2009)	*	*	*	*	*	
Croatia (Harvey, Kalanj et al. 2004)					*	
Cuba (Sixto 2002, Whiteford and Branch 2008)			*			
Cypriot (Pallari *et al.*, 2020)				*		
Denmark (Setlhare 2016)	*	*	*	*		*
Ecuador (Jimenez and San Sebastián 2021)				*		
England (Blumenthal and Dixon 2012)	*	*	*	*		*
Ethiopia (Bradley *et al.*, 2012)	*	*	*	*		*
Finland (Saarivirta, Consoli *et al.* 2012, Tynkkynen *et al.*, 2016)	*	*		*		
Georgia (Gamkrelide *et al.*, 2002, Gotsadze *et al.*, 2005)	*	*				*
Greece (Tragakes and Polyzos 1998, Tountas, Karnaki et al. 2002)	*	*	*			*
India (Ghosh 2014, Rahman, Angeline et al. 2014)	*	*	*	*	*	
Iran (Shadpour 2006, Lankarani 2012, Esmailzadeh *et al.*, 2013, Lankarani *et al.*, 2013, Asaei 2014, Heshmati and Joulaei 2016)	*	*	*	*	*	*
Kazakhstan (Organization and UNICEF 1997, Abzalova, Wickham et al. 1998)	*	*	*	*		*
Kosovo (Buwa and Vuori 2006, Percival and Sondorp 2010)	*	*	*	*		*
Lithuania (Liseckienė 2009, Buivydiene, Starkiene et al. 2010)	*	*	*	*		
Malaysia (Yu, Whynes et al. 2011)		*				
Mexico (Frenk, González-Pier et al. 2006, van Weel, Turnbull et al. 2016)	*	*		*		*
New Zealand (Gauld 2001, Tenbensel 2008)	*	*	*	*		*
Philippines (Obermann, Jowett et al. 2008)	*	*		*		
Poland (Mokrzycka, Kowalska-Bobko et al. 2016)				*		
Portugal (Biscaia and Heleno 2017)	*	*	*	*	*	*
Romanian (Bara *et al.*, 2002)	*	*				
Slovenian (Vab 1995)	*	*				
South Africa (Benatar 2004, Van Pletzen *et al.*, 2013, Okoronkwo, Onwujekwe *et al.* 2014, Schneider, English et al. 2014)	*	*		*		
South America (Ramírez *et al.*, 2011, Acosta Ramírez *et al.*, 2016)	*		*	*		*
Spain (Larizgoitia and Starfield 1997)	*		*	*		
Sweden (Spak and Andersson 2008, Forsberg 2018)	*	*		*		
Turkey (Tatar and Kanavos 2006, Yasar 2011, Jadoo, Aljunid *et al.* 2014, Hone, Gurol-Urganci et al. 2017)	*	*	*	*	*	*
UAE (Koornneef, Robben et al. 2012, Koornneef, Robben et al. 2017)	*	*		*	*	*
Uganda (Tashobya 2004)	*	*	*	*		

*Notes*: * indicates that the country’s reforms concerned the building block.

### PHC service delivery

PHC reforms have predominantly aimed to reduce health disparities by enhancing access to health services, minimizing variations in care quality, and optimizing resources across both public and private sectors. A common approach adopted in numerous countries has involved establishing PHC service units in both urban and rural settings to bridge access gaps (Gauld 2001; Yip and Hsiao 2009; Hu *et al.*, 2011; Bradley *et al.*, 2012; Ghosh 2014; Schneider, English, et al. 2014). Additionally, several reforms have emphasized expanding the cadre of family physicians, who function as gatekeepers within health systems (Tatar and Kanavos 2006; Mokrzycka, Kowalska-Bobko, et al. 2016). Governments have also sought to increase competitiveness in service delivery by encouraging private sector participation and volunteer-based health services (Bastías, Pantoja, et al. 2008; Ghosh 2014; Forsberg 2018). To enable more holistic patient management, a key objective has been the integration of services such as mental health and rehabilitative care (Schneider *et al.* 2014). However, in some contexts, PHC systems remain fragmented, with poorly coordinated services that compromise patient outcomes. To better address local health needs, community engagement has been promoted as a critical component (Bastías *et al.* 2008; Forsberg 2018). Many countries recognize PHC as a cost-effective model for health service delivery (Tatar and Kanavos 2006; Mokrzycka, *et al.* 2016), yet workforce shortages and limited funding continue to impede effective service provision in certain regions (Ghosh 2014).

### PHC financing

Many countries have increasingly adopted integrated payment models and performance-based payment systems to incentivize healthcare providers financially (Hu et al., 2011; Lankarani 2012; Harris *et al.*, 2015; Biscaia Heleno 2017; Pu *et al.*, 2020). Recent reforms in healthcare financing policies have primarily focused on enhancing financial commitments from various governmental bodies (Tountas *et al.*, 2002; Bastías *et al.*, 2008; Yip Hsiao 2009; Baum *et al.*, 2013; Asaei 2014). Almost all reviewed financing reforms have sought to implement measures aimed at expanding health insurance coverage and mitigating catastrophic expenditures, particularly for marginalized populations, thereby advancing the pursuit of Universal Health Coverage (UHC) (Frenk *et al.*, 2006; Unger *et al.*, 2008; Buivydiene *et al.*, 2010; Yu *et al.*, 2011; Schneider *et al.*, 2014; Heshmati Joulaei 2016). However, the allocation of financial resources within many primary health care systems, especially in rural and underserved areas, remains a significant challenge (Hu et al., 2011; Baum *et al.*, 2013). Furthermore, in numerous countries, financing models are heavily dependent on political will and external funding, raising concerns about their long-term sustainability (Lankarani 2012; Tountas *et al.*, 2002).

### PHC health workforce

Our analysis indicates that most reforms have aimed to enhance the training of personnel for primary health care while promoting the equitable distribution of qualified health professionals across various levels of health service delivery (Tashobya 2004; Tatar Kanavos 2006; Yip Hsiao 2009). Specific interventions have been designed to improve and expand training services by reforming medical education and implementing capacity-building programmes for primary health care workers, family physicians, community health professionals, and health graduate students. These initiatives are essential for equipping professionals to meet the evolving demands of clients and health systems, enabling them to provide comprehensive health services that encompass health promotion, prevention, improvement, and rehabilitation (Larizgoitia Starfield 1997; Hotchkiss *et al.*, 2005; Yasar 2011; Rahman *et al.*, 2014; Setlhare 2016). Nevertheless, critical issues such as shortages of well-trained primary health care personnel, inequitable distribution of the workforce, burnout, and job dissatisfaction must be urgently addressed to ensure the effectiveness and sustainability of primary health care systems.

### PHC leadership and governance

Effective leadership and governance are pivotal in shaping robust PHC systems. Most reforms have prioritized initiatives aimed at enhancing service quality and ensuring accountability among health consumers and providers. This has been achieved through the implementation of comprehensive legislation, regulations, and standards designed to improve management, planning, healthcare purchasing, and monitoring and evaluation (Frenk *et al.*, 2006; Bastías *et al.*, 2008). Key strategies include the decentralization of administrative and regulatory functions, alongside the delivery of health services across various levels (Gauld 2001; Obermann *et al.*, 2008). Furthermore, promoting public-private partnerships, fostering multisectoral cooperation, strengthening the institutional capacities of Ministries of Health, and encouraging social participation have been critical. Political and religious commitments to provide financial, material, and human resources are also essential for enhancing health service delivery (Ghosh 2014; van Weel *et al.*, 2016). Despite these advancements in leadership and governance within PHC, challenges remain. Effective coordination, community engagement, and equitable access to health services must be prioritized to fully realize the potential of these reforms.

### PHC medical products, vaccines, and technologies

Recent reforms have focused on the introduction and improvement of essential medicines (Harvey *et al.*, 2004; Hu *et al.*, 2011; Koornneef *et al.*, 2012), as well as the establishment of guidelines for drug production, prescription, pricing, and supply (Tenbensel 2008; Yip Hsiao 2009; Koornneef *et al.*, 2017). Additionally, efforts to enhance infrastructure and increase the availability of essential equipment at the PHC level have been emphasized (Hotchkiss *et al.*, 2005; Atun *et al.*, 2007; Parfitt 2009; Yasar 2011; Kuchenbecker Polanczyk 2012). These interventions have successfully controlled prescription drug price inflation, facilitated service provision, ensured access to essential drugs, expanded vaccine coverage, improved the quality of prescribing practices by physicians, and updated leading health services. However, despite these efforts to improve access to medical products, vaccines, and technologies, inequities persist. During crises or pandemics, disruptions in supply chains have exacerbated vulnerabilities in access to essential medicines and vaccines (Harvey *et al.*, 2004; Hotchkiss *et al.*, 2005).

### PHC information

Several PHC information reforms have focused on enhancing health management through the establishment and implementation of advanced information systems (Gauld 2001; Hotchkiss, Piccinino, et al. 2005; Xu *et al.*, 2007; Blumenthal and Dixon 2012; Koornneef, Robben, et al. 2012), the upgrading of health information systems (Frenk, González-Pier, et al. 2006; Yasar 2011), and the adoption of electronic health records (Tountas, Karnaki, et al. 2002; Blumenthal and Dixon 2012). Additionally, information technology networks have been established to facilitate seamless data exchange (Setlhare 2016). These initiatives have collectively improved access to comprehensive health data, bolstered health information protection, enabled access to electronic records, facilitated indicator evaluation through electronic records, and supported continuous and effective service monitoring, thus preserving and utilizing health records to enhance patient care. Enhanced access to information has also provided policymakers with a robust basis for making informed decisions that aim to improve health outcomes (Yasar 2011; Setlhare 2016). With the digitalization of health data, however, concerns around data privacy and security have become increasingly prominent (Blumenthal and Dixon 2012; Yasar 2022). Furthermore, equitable access to health information remains a critical issue, necessitating careful consideration in the design and implementation of PHC reforms to ensure inclusivity in both availability and use (Frenk *et al.* 2006; Tountas *et al.* 2002).

Enhanced access to information has also provided policymakers with a robust basis for making informed decisions that aim to improve health outcomes (Yasar 2011; Setlhare 2016). With the digitalization of health data, however, concerns around data privacy and security have become increasingly prominent (Blumenthal and Dixon 2012; Yasar 2022). Furthermore, equitable access to health information remains a critical issue, necessitating careful consideration in the design and implementation of PHC reforms to ensure inclusivity in both availability and use (Frenk *et al.* 2006; Tountas *et al.* 2002).

## Discussion

We systematically reviewed studies on interventions aimed at reforming PHC systems, assessing their objectives and outcomes using the WHO Health System Framework as a reference. Our findings indicate that most policy reforms focused on expanding service delivery, improving financing, and providing essential medicines and technology to meet the health care needs of populations.

In response to the challenges facing PHC, numerous countries have implemented targeted reforms to strengthen their health systems (Mulligan and Castañeda 2018). The specific context of each country, alongside the priorities set by policymakers and planners, has influenced the nature and scope of these reforms. For example, in the United States, the Affordable Care Act was enacted to expand health insurance coverage and improve access to care (Gaffney and McCormick 2017). In the United Kingdom, the National Health Service (NHS) has undergone multiple reforms aimed at increasing system efficiency and enhancing patient outcomes (Alderwick and Dixon 2019). Iran, likewise, has introduced several notable PHC reforms over recent decades, including the NHS Corps Law (1964), the establishment of the National Health Network (1985), the implementation of the Rural Family Physician programme (2005) (Shirjang *et al.* 2020), and the Health Transformation Plan (2014) (Nasseri *et al.* 1991; Heshmati and Joulaei 2016; Takian *et al.* 2015; Doshmangir *et al.* 2019). These initiatives reflect a range of strategies adapted to meet local needs and priorities in strengthening PHC delivery. Reform efforts are often shaped by each country’s specific challenges, political environment, and economic conditions (Chernichovsky 2019). In Western Europe, for example, health systems tend to focus on equitable access, public financing, and comprehensive coverage (Van Loenen *et al.* 2016), whereas the U.S. relies more on a private, market-driven health care system (Mulligan and Castañeda 2018). In Latin America, reforms emphasize universal health coverage and strengthening primary care, as seen in Brazil’s Integrated Health System (SUS) and Mexico’s Seguro Popular programme (Arredondo *et al.* 2018).

Despite these reforms, some critical areas are often neglected. For example, prevention and primary care do not always receive the attention they deserve, and mental health is still widely overlooked in many countries (McConville and Hooven 2021; Stumbo *et al.* 2018). Health inequities, driven by socioeconomic or geographic disparities, remain inadequately addressed in many systems (Oberg *et al.* 2016). In terms of service delivery, many countries have prioritized expanding PHC services in both urban and rural areas. This has often involved collaboration between public and private providers, especially in nations like New Zealand (Gauld 2001), China (Yip and Hsiao 2009), South Africa (Schneider *et al.* 2014), and Mexico (Frenk *et al.* 2006). The Family Physician programme has proven effective in delivering comprehensive care at a low cost, which has made it a central feature of many health reforms (Yordy and Vanselow 1994; Shirjang *et al.* 2020). Countries such as Turkey, Spain, Kosovo, Sweden, Australia, and Iran have all focused on improving PHC as a critical element of their reform efforts (Tatar and Kanavos 2006; Larizgoitia and Starfield 1997; Percival and Sondorp 2010; Forsberg 2018; Heshmati and Joulaei 2016).

In PHC delivery, private sector has a larger role. Countries like Sweden, Australia, and Iran have harnessed the capacities of non-governmental organizations (NGOs) and private providers to address health care challenges, particularly in underserved areas (Palmer 2000). These efforts have contributed to the progress toward Universal Health Coverage (UHC), as evidenced by increased access to PHC in countries such as China, Turkey, Sweden, Uganda, Brazil, and Iran (Yip and Hsiao 2009; Hone *et al.* 2017; Tashobya 2004; Shadpour 2006). In some countries like Sweden, voluntary providers, such as NGOs, community health workers, and other non-profit entities provides PHC services such as prevention, promotion and chronic disease management, particularly in remote and underdeveloped areas.

Health financing reforms have been another major focus, with many countries revising service packages and reducing out-of-pocket expenses to protect low-income groups (Kutzin 2013; Kieny and Evans 2013). Performance-based financing mechanisms have been used to encourage preventive care, especially in remote areas, as seen in countries like China, Turkey, Canada, and Iran (Tang *et al.* 2008; Heshmati and Joulaei 2016). Expanding social health insurance has also been a successful strategy in countries such as China, Chile, South Africa, Mexico, and Iran to improve access and reduce the financial burden of medical expenses (Meessen *et al.* 2011).

Finally, evidence highlights the importance of strong political leadership and sustained financial investment in ensuring successful health system reforms (Faye *et al.* 2012). Countries that implement reforms with robust monitoring systems tend to experience fewer challenges and better health outcomes (Griswold *et al.* 2018).

## Strengths and Limitations of the study

Given the broad scope of the subject, our study was restricted to peer-reviewed publications, excluding grey literature, which may have limited the comprehensiveness of our review. However, by incorporating a wide range of study designs, including qualitative, quantitative, and mixed-methods research, we were able to provide a thorough and nuanced analysis of the available evidence.

## Conclusion

Despite substantial progress towards UHC, health systems globally continue to face persistent challenges that threaten sustainable health development. Many of the reviewed reforms in PHC and public health have focused on advancing UHC by expanding service delivery in both rural and urban areas, establishing and enhancing service provider networks, increasing budget allocations to public health and PHC, and expanding family physician programmes, alongside efforts to build the capacity of PHC workers. Achieving sustainable progress towards UHC requires carefully planned, evidence-based policy interventions that strengthen the PHC system, with a focus on improving governance and leadership. While cross-national learning is essential, reform strategies must be tailored to the specific national context through the application of contextually relevant models.

## Supporting information

Shirjang et al. supplementary material Shirjang et al. supplementary material

## Supplementary material

The supplementary material for this article can be found at https://doi.org/10.1017/S1463423625000271
