# Supplementary material for: Primary health care reforms: a scoping review
Source: Prim Health Care Res Dev. 2025 Aug 18;26:e74. doi: 10.1017/S1463423625000271 (PMC12455359; doi:10.1017/S1463423625000271)
Supplement: Shirjang et al. supplementary material [file S1463423625000271sup001.docx]

**SUPPLEMENTARY MATERIAL**

**APPENDIX 1: Preferred Reporting Items for Systematic reviews and Meta-Analyses extension for Scoping Reviews (PRISMA-ScR) Checklist**

| **SECTION** | **ITEM** | **PRISMA-ScR CHECKLIST ITEM** | **REPORTED ON PAGE #** |
| --- | --- | --- | --- |
| **TITLE** | | | |
| Title | 1 | Identify the report as a scoping review. | 1 |
| **ABSTRACT** | | | |
| Structured summary | 2 | Provide a structured summary that includes (as applicable): background, objectives, eligibility criteria, sources of evidence, charting methods, results, and conclusions that relate to the review questions and objectives. | 1 |
| **INTRODUCTION** | | | |
| Rationale | 3 | Describe the rationale for the review in the context of what is already known. Explain why the review questions/objectives lend themselves to a scoping review approach. | 2 |
| Objectives | 4 | Provide an explicit statement of the questions and objectives being addressed with reference to their key elements (e.g., population or participants, concepts, and context) or other relevant key elements used to conceptualize the review questions and/or objectives. | 2 |
| **METHODS** | | | |
| Protocol and registration | 5 | Indicate whether a review protocol exists; state if and where it can be accessed (e.g., a Web address); and if available, provide registration information, including the registration number. | 3 |
| Eligibility criteria | 6 | Specify characteristics of the sources of evidence used as eligibility criteria (e.g., years considered, language, and publication status), and provide a rationale. | 3 |
| Information sources* | 7 | Describe all information sources in the search (e.g., databases with dates of coverage and contact with authors to identify additional sources), as well as the date the most recent search was executed. | 3 |
| Search | 8 | Present the full electronic search strategy for at least 1 database, including any limits used, such that it could be repeated. | 3 |
| Selection of sources of evidence† | 9 | State the process for selecting sources of evidence (i.e., screening and eligibility) included in the scoping review. | 3 |
| Data charting process‡ | 10 | Describe the methods of charting data from the included sources of evidence (e.g., calibrated forms or forms that have been tested by the team before their use, and whether data charting was done independently or in duplicate) and any processes for obtaining and confirming data from investigators. | 3 |
| Data items | 11 | List and define all variables for which data were sought and any assumptions and simplifications made. | 3 |
| Critical appraisal of individual sources of evidence§ | 12 | If done, provide a rationale for conducting a critical appraisal of included sources of evidence; describe the methods used and how this information was used in any data synthesis (if appropriate). | 3 |
| Synthesis of results | 13 | Describe the methods of handling and summarizing the data that were charted. | 3 |
| **RESULTS** | | | |
| Selection of sources of evidence | 14 | Give numbers of sources of evidence screened, assessed for eligibility, and included in the review, with reasons for exclusions at each stage, ideally using a flow diagram. | 4 |
| Characteristics of sources of evidence | 15 | For each source of evidence, present characteristics for which data were charted and provide the citations. | 4 |
| Critical appraisal within sources of evidence | 16 | If done, present data on critical appraisal of included sources of evidence (see item 12). | 5 |
| Results of individual sources of evidence | 17 | For each included source of evidence, present the relevant data that were charted that relate to the review questions and objectives. | 4,5 |
| Synthesis of results | 18 | Summarize and/or present the charting results as they relate to the review questions and objectives. | 4,5 |
| **DISCUSSION** | | | |
| Summary of evidence | 19 | Summarize the main results (including an overview of concepts, themes, and types of evidence available), link to the review questions and objectives, and consider the relevance to key groups. | 5-7 |
| Limitations | 20 | Discuss the limitations of the scoping review process. | 7 |
| Conclusions | 21 | Provide a general interpretation of the results with respect to the review questions and objectives, as well as potential implications and/or next steps. | 8 |
| **FUNDING** | | | |
| Funding | 22 | Describe sources of funding for the included sources of evidence, as well as sources of funding for the scoping review. Describe the role of the funders of the scoping review. | 8 |

**Appendix S2: Search strategy**

| **Search strategy** | **Number of articles** | **Search engine** |
| --- | --- | --- |
| ti("phc") OR ti("primary health care") AND ti("revise") OR ti("reform") OR ti("policy") AND ti("intervention") | 204 | Proquest |
| ('phc':ab,kw,ti OR 'primary health care':ab,kw,ti) AND ('reform*':ab,kw,ti OR 'revise*':ab,kw,ti OR 'policy':ab,kw,ti OR 'intervention':ab,kw,ti) AND [article]/lim AND [humans]/lim AND [english]/lim AND [abstracts]/lim | 1004 | Embase |
| TOPIC AND ABSTRACT: ("PHC" OR "primary health care") AND TOPIC AND ABSTRACT: ("intervention" OR "revise*" OR "reform*" OR "policy*") | 290 | Science Direct |
| ( TITLE-ABS-KEY ( "PHC" )  OR  TITLE-ABS-KEY ( "primary health care" )  AND  TITLE-ABS-KEY ( "revise*" )  OR  TITLE-ABS-KEY ( "reform*" )  OR  TITLE-ABS-KEY ( "policy" )  OR  TITLE-ABS-KEY ( "intervention*" ) )  AND  ( LIMIT-TO ( SRCTYPE ,  "j" ) )  AND  ( LIMIT-TO ( DOCTYPE ,  "ar" ) )  AND  ( LIMIT-TO ( LANGUAGE ,  "English" ) )  AND  (  LIMIT-TO ( ACCESSTYPE(OA) ) ) | 772 | Scopus |
| allintitle:"PHC" OR "primary health care" AND "revise *" OR "reform *" OR "policy*" OR "intervention" | 442 | Google Scholar |
| (((("primary health care"[Title/Abstract] OR "phc"[Title/Abstract]) AND "revise*"[Title/Abstract]) OR "reform*"[Title/Abstract]) OR "policy*"[Title/Abstract]) OR "intervention*"[Title/Abstract] AND (Clinical Trial[ptyp] AND "loattrfree full text"[sb] AND "humans"[MeSH Terms] AND English[lang] AND systematic[sb] AND (jsubsetn[text] OR medline[sb])) | 770 | PubMed |
|  | 3482 |  |

**Appendix S3: Review of primary health care reforms in different countries (financing and human resources)**

| **Human Resources** | | **Financing** | | **References**  **Country** |
| --- | --- | --- | --- | --- |
| **Outcome/impacts** | **Focus of intervention** | **Outcome/impacts** | **Focus of intervention** |  |
|  | Transfer GP^1^ to PCO^2^s | Increase equity in access to services  Citizens who earn more than a certain level of income pay all expenses | Per capita health distribution depending on the level of deprivation  Allocation of resources based on regional need | New Zealand (Gauld 2001, Tenbensel 2008) |
| Expanding public health services | Training of rural health care providers  Motivating to work in low-income areas | Reducing the cost of patient care  Accelerating the process of allocating resources  Break the financial relationship between the patient and the doctor  Reducing out-of-pocket payments in urban areas  Increasing the income of health workers in poor areas  Financial pressure in low-income groups in rural areas  Decreasing Kakwani index | Reforms in health care financing policies, i.e., by increasing the share of organized financing (government and social fund) in total health expenditures  Implementation of New RCMS^3^ to sustain public health care costs  Amending provider payment mechanism | China (Xu, Wang et al. 2007, Tang, Meng et al. 2008, Yip and Hsiao 2009, Hu, Zou et al. 2011, Sun, Chai et al. 2014, Lin, Sun et al. 2015) |
|  |  | Expanding health insurance coverage | Increase government health costs (National and municipal governments cover primary care, making it free at the point of delivery)  Reforms in health insurance coverage (implementation of dual health care system under which its citizens can voluntarily opt for coverage by either the public National Health Insurance Fund or any of the country’s private health insurance companies) | Chile  (Bastías, Pantoja et al. 2008, Unger, De Paepe et al. 2008, Cornejo-Ovalle, Brignardello-Petersen et al. 2015) |
|  |  | Expanding relatively fair and stable coverage | Introducing health insurance with sufficient financial resources | South Africa (Benatar 2004, Van Pletzen, Zulliger et al. 2013, Schneider, English et al. 2014, Le Roux, Le Roux et al. 2015) |
|  |  | Increasing health care budget  Appling subsidies from rich to poor | New Insurance Plan (NHI)^4^,  Financial reform | Malaysia (Yu, Whynes et al. 2011) |
|  | Reforms in medical education | Reducing catastrophic health costs for poor people | Per capita distribution to the states  Reducing public health costs | India (Ghosh 2014, Rahman, Angeline et al. 2014)  NRHM^5^ |
|  |  | Increasing justice between provinces in financing  Reducing catastrophic costs for disadvantaged individuals and groups  Upgrading all financial protection indicators  Lack of total allocation of resources at the state level | Expanding public health insurance for the entire population  Implementing protective programs against catastrophic costs  Decentralization for funding essential services  Increasing public health costs | Mexico (Frenk, González-Pier et al. 2006, van Weel, Turnbull et al. 2016)  SSPH^6^ |
|  |  | High rate of registration in health insurance programs | Compulsory health insurance | UAE (Koornneef, Robben et al. 2012, Koornneef, Robben et al. 2017) |
|  |  | Reducing access to public finance | Decentralization in primary care financing  Implementing per capita payment strategies | Georgia (Gamkrelide, Atun et al. 2002, Gotsadze, Zoidze et al. 2005) |
| Improving the quality of care | The primary providers of health care are primarily physicians | Increasing employee satisfaction and motivation | Reforming of provider payment mechanism (the method of integrated payments mainly per capita, while examining the types of performance-based payment methods - based on service value | America (Blumenthal and Dixon 2012, Lankarani 2012) |
| The rapid expansion of the health workforce | Increasing the number of general practitioners and specialists | Public health insurance coverage  Reducing out-of-pocket payments | Reinforcing payment to the executive system  Increasing the government health expenditures | Turkey (Tatar and Kanavos 2006, Yasar 2011, Hone, Gurol-Urganci et al. 2017) |
|  |  | Increasing the cost of health care for SES^7^ groups higher than low SES  Paying out of pocket above  The low government budget for health care | Building a new health care system focusing on reforming quality and cost for health care | Philippines (Obermann, Jowett et al. 2008) |
|  |  | Expansion of social services  Supplementary amounts in the form of per capita according to the needs of municipalities (instead of allocating fixed subsidies) | Allocating additional amounts to municipalities  Major funding through government, employers and taxes | Finland (Saarivirta, Consoli et al. 2012, Tynkkynen, Chydenius et al. 2016) |
| Incorporating family physicians into the system  Multi-professional activity | Family physicians training  Teamwork training |  |  | Spain (Larizgoitia and Starfield 1997) |
| Deployment of family doctors in the health centre | Family physicians training | Increasing volunteer investors  Inadequate funding of health care by the government | Support volunteer investors for HSR^8^ | Kosovo (Buwa and Vuori 2006, Percival and Sondorp 2010) |
|  |  | Private sector activity in privileged areas  No increase in health care costs | Payment to both public and private sectors in the form of per capita | Sweden  (Spak and Andersson 2008, Forsberg 2018) |
| Professional independence of physicians within the health care system | Defining the main role of general practitioners  Strengthening the important role of professionals and professional disciplines in primary health care | Establish an independent hospital fund  Use of General Patient Standard (SWPE)^9^ criteria based on care  Increased willingness to provide remote care in remote villages and areas | Responsibility for financing with the Commonwealth of Independent States  Incentive-based budgeting system  Paying more for service in rural and remote areas | Australia  (Baum, Freeman et al. 2013, Baum, Freeman et al. 2016) |
| Increasing physician  Familiarity of health workers with ECG, sampling, acupuncture  Employing graduates of various disciplines to provide services  Creating strong teams | Estimation and training of required physicians   Comprehensive training (practical practice) Family physician  Empowering health workers  Strengthen other health disciplines  Strengthen the interaction of family physicians and staff | Affordable and efficient health care system and free primary care | Public health financing with higher taxes  Increasing health costs | Denmark (Setlhare 2016) |
| Problem-solving capacity (managers-personnel (HEW)^11^ | Training of health workers and counselling at the place of service  Improving group relationships (between employees and officials) |  | Increasing financial resources  delegation of authority and spending part of the budget to PHCU^10^ | Ethiopia  (Bradley, Byam et al. 2012) |
|  | Intensive clinical training program for service providers | Motivating to improve performance  Rising per capita government spending on health  Increasing out-of-pocket payments | Financing by the United States Agency for International Development (USAID)^13^  New reward system for doctors | Armenia (Grigoryan 2005)  Armenian Urban Transfer Project (ASTP)^12^2015-2005 |
| Improving the quality and sustainability of services | Rehabilitation of Family Physician Doctors  Rehabilitation of nurses with European standards  Supplementary course for family health nurses and midwives | Positive impact on employee morale and motivation | Increasing the income of health care workers | Commonwealth of Independent States of Central Asia (Parfitt 2009)  Tajikistan, Uzbekistan,  Azerbaijan, Georgia, Kyrgyzstan |
| Increasing the number of visits per person per year, especially general practitioners  Increasing people's satisfaction | Approval of licensing for private sector participation (exploring the possibility of a contract between different providers (public-private) and national insurance)  Providing family physician services | Eliminating budget payments  Public coverage of the population | Introducing the National Health Insurance Plan through Taxation | Slovenian (Vab 1995) |
| Proper organization of human resources | Decentralized human resource management | Releasing resources with the aim of spending for the vulnerable people | Payment by client services  (Social prepayment) | Uganda (Tashobya 2004) |
| Improved services quality | Training providers (doctors and nurses) in line with clinical guidelines | Improve budgeting  Improved financing  Improving plan | Health financing reforms  Settling financial policies based on national PHC guidelines | Albania (Hotchkiss, Piccinino et al. 2005)  PHRplus^14^  2000-2004 |
| Improved human resources | The geographical allocation of health teams equally  Empowering interdisciplinary teams |  |  | South America  (Acosta Ramírez, Giovanella et al. 2016) |
|  |  | Increase insurance coverage  High levels of paying out of pocket and under the table payments | Introducing a new social insurance | Romanian  (Bara, Van den Heuvel et al. 2002) |
| Provision of efficient human resources  Existence of professional doctors and nurses | knowledge management  Strengthening educational activities  Proper organization of human resources | Financial sustainability of primary care | Decentralization in the distribution of primary health care resources and delegation of budget distribution to regions | Brazil (Almeida, Travassos et al. 2000, Kuchenbecker and Polanczyk 2012, Soranz, Pinto et al. 2016)  RCAPS^15^  2009-2015 |
| Strengthened interprofessional health relationships | Providing extra training to physicians to work as family physicians  Encourage teamwork  Improving professional identity | Improved PHC funding | Promoting charitable donations to fund PHC | Bosnia and Herzegovina (Atun, Kyratsis et al. 2007)  FaMI Project^16^  2004-2007 |
| Improved motivation among primary health care workers  Improved accountability | Communicate and provide service providers  Reforming human resource targeting efficacy of managers to improve primary health care management  Promoting staff participation in reform policy | Improving motivation among primary health care workers  Increased interest in primary care  Logical and creative use of doctors' resources and more substantial commitment | Performance-based per capita payment | Kazakhstan (Organization and UNICEF 1997, Abzalova, Wickham et al. 1998) |
| Increased the independence and flexibility of workforce  Availability of voluntary health service providers and organized multi-disciplinary teams | Investing in human resource development  Encouraging teamwork | Motivate to provide service  Creating innovation capacity in the health system | Distinctive and performance-related payment. | Portugal (Szczygieł, Pinto Lima et al. 2011, Biscaia and Heleno 2017)  CSP^17^ 2005 |
| Improved service quality | Creating interprofessional teams to provide services  Teaching multi-disciplinary working group activities in the form of theory and practice | Deleting the FFS^18^ payment  Increase the interest of service providers | Paying in the form of Per capita or in the mix form | Canada  (Tenbensel 2008, Oandasan, Conn et al. 2009, Strumpf, Levesque et al. 2012, Harris, Green et al. 2015) |
| Expanded use of primary care educational scenarios in the program  Improved curriculum for medical science graduates | Training health professionals  Comprehensive medical concepts training (health promotion, prevention, improvement and rehabilitation)  Inclusion of a family physician in the medical curriculum |  |  | Cuba (Sixto 2002) |
| Improved inter-professional communication | Formation of primary care group (PCG)^20^  Formation of primary care teams (PCT)^21^ | Funding for the health care organization  Reducing drug costs  Increasing administrative costs | NHS^19^ financing by the government  and part of the administrative costs of local health services by local taxes | England (Blumenthal and Dixon 2012) |
| Increased number of trained family doctors  Increased number of specialist doctors in small cities  Improved teamwork  The increased workload of personnel | Eligibility of family physicians (retraining and retraining)  Encouraging doctors to work as family physicians  Distribution of specialist doctors to deprived areas | Payments in the form  - Per capita  - Additional costs for effective services (preventive services) and  - Beneficial (early detection of cancer).  Increasing doctors' dissatisfaction because of payment | Providing a mixed insurance payment system based on social insurance  Establishment of State and Regional Insurance Fund (TSF)^22^ | Lithuania  (Liseckienė 2009, Buivydiene, Starkiene et al. 2010) |
| Adequate workforce supply | Providing trained personnel | Removing unofficial payments | Merge social funds and create an integrated financing system   ODIPY^25^ | Greece (Tragakes and Polyzos 1998, Tountas, Karnaki et al. 2002)  PASOK^23^ 2000  SCUC2^24^ 2017 |
| Availability of multi-disciplinary teams (general practitioner/family, paediatrician, nurse, health supervisor and social workers) | Human resource development  Implementation of a short-term retraining program for general practitioners  Inter-professional work training to health personnel  Review of general practitioners training course | Improving health  Public coverage and strengthening the effectiveness, efficiency and flexibility of the health system | EU ^26^ funding through an agreement between the European Commission and WHO / Europe |  |
| Trained personnel required for the first level of service delivery  Educated family physicians to provide comprehensive health services (health promotion, resuscitation, recovery and rehabilitation)  Increased number of psychologists and nutritionists in the PHC level | Establishment of welfare schools  Reforms in medical education  Strengthening the role of graduates in other health-related fields in primary health care | Increasing staff  Motivation  Increasing services  Increasing the capacity of health services | Performance-based payment  Increasing the share of health from the public budget | Iran  (Shadpour 2006, Lankarani 2012, Esmailzadeh, Rajabi et al. 2013, Asaei 2014, Malekafzali 2014, Heshmati and Joulaei 2016, Doshmangir, Moshiri et al. 2020) |

**Note:** Abbreviations:

1. GP: General practitioner
2. PCOs: Primary Care Organizations
3. RCMS: Rural Cooperative Medical System
4. NHI: National Health Insurance
5. NRHM: National Rural Health Mission
6. SSPH: System of Social Protection in Health
7. SES: Socio-economic status
8. HSR: Health sector reforms
9. SWPE: Standardized Whole Patient Equivalent
10. PHCU: Primary Health Care Units
11. HEW: Health Extension Worker
12. ASTP: Armenia Social Transition Program
13. USAID: United States Agency for International Development
14. PHRplus: Partners for Health Reform plus
15. RCAPS: Themes and Reform of Primary Health Care
16. FaMI Project: has been implementing Family Medicine ( in various regions)
17. CSP: Portuguese primary health care
18. FFS: Fee-for-service
19. NHS: National Health System
20. PCG: Primary Care Groups
21. PCT: Primary Care Trusts
22. TSF: Territorial Sickness Funds
23. PASOK: social-democratic political party in Greece
24. SCUC2: Strengthening Capacity for Universal Coverage, Phase 2
25. ODIPY: Organismos Diahirisis Poron Ygias
26. EU: the European Union

**Appendix S4: Review of primary health care reforms in different countries (health service delivery and leadership/governance)**

| **Governance** | | **Health Service Delivery** | | **References**  **Country** |
| --- | --- | --- | --- | --- |
| **Outcome/impacts** | **Intervention** | **outcome** | **Intervention** |  |
| Improved community health  Regional Boards of Directors often plan and provide health services for specific areas. | Changing the organizational structure (returning to a more decentralized structure based around 21 District Health Boards  Primary Care Organizations (PCOs) | Expanding the provision of primary health care  Reducing unnecessary hospitalizations | Create PHO ^1^in each DHB^2^ | New Zealand (Gauld 2001, Tenbensel 2008) |
| The rapid development of the health sector  Increased Public insurance coverage  Private sector resistance due to the expansion of public health services  Enabled by top-down procedures, counties were able to bottom-up tailor the content of best-practice innovations to fit local needs | Strengthen public health performance and services  Increasing the role of the private sector in the health system  Hybrid approaches reside—from bottom-up to top-down—has two dimensions: a content dimension and a procedural dimension | Increasing coverage of NCMS^3^  Significant reduction in maternal and child mortality Improving Access to Primary Health Care | Improving access and quality of primary health care | China  (Xu, Wang et al. 2007, Tang, Meng et al. 2008, Yip and Hsiao 2009, Hu, Zou et al. 2011, Sun, Chai et al. 2014, Lin, Sun et al. 2015, Klundert, de Korne et al. 2020) |
| Improved performance monitoring capacity in the health market (apart from providers)  Provide additional resources needed for financial reform  Improved private sector regulations  Encouraging decision-making decentralization and shared responsibility | Adoption of the Health Management Law  Financing act from the state budget  Private Health Law  Collaborative Access to Health with Explicit Guarantees (AUGE)^5^ | Increasing the use of health services  Increasing the use of inpatient and outpatient services by low SES ^4^groups  Unequal use of services between different SES groups | Private sector activity license in the health care system in addition to specialized care | Chile  (Bastías, Pantoja et al. 2008, Unger, De Paepe et al. 2008, Cornejo-Ovalle, Brignardello-Petersen et al. 2015) |
| Rapid response of the government to systematic changes | Providing and regulating health insurance | Horizontal expansion of public services  The decline of the private sector | Providing services by the government | South Africa (Benatar 2004, Van Pletzen, Zulliger et al. 2013, Schneider, English et al. 2014, Le Roux, Le Roux et al. 2015) |
| improve the institutional architecture of the NHS | adoption of the Integral Health Model and the Integral Public Health Network |  |  | Ecuador(Quizhpe, San Sebastian et al. 2020, Jimenez and San Sebastián 2021) |
| Improving health indicators | introduction of primary care driven national healthcare system |  |  | Cypriot(Pallari, Samoutis et al. 2020) |
| Improving some health indicators  Centralized supply and distribution system | Decentralization  Inter-sectorial cooperation  Integrated health policies | Increased use of health care by low and rural SES^4^  Improving the quality of government services  Use of private sector care by medium/high SES^4^  Lack of attention to urban population | Provide PHC and private health care | India (Ghosh 2014, Rahman, Angeline et al. 2014) |
|  | Designing monitoring and evaluation systems  Advancing HSR^6^ with the participation of state funds | Improving effective coverage for interventions  Improving the quality of services and health indicators  Improving health participation  Reduction in coverage differences between SES groups  Increasing the efficiency and effectiveness of health care  Health gaps between social and economic groups and different states | Demand-based services  Provide primary care for uninsured people | Mexico  (Frenk, González-Pier et al. 2006, van Weel, Turnbull et al. 2016) |
| Strengthen the centralized monitoring system | Introduction of mandatory private  health insurance,  and separation of planning and regulatory responsibilities  from provider functions | Increasing the number of health facilities  Intensifying competition between providers  Unequal improvement in quality and utilization of services | Expanding the use of private sector providers | UAE (Koornneef, Robben et al. 2012, Koornneef, Robben et al. 2017) |
|  |  | Increasing service usage  Reducing self-medication  Inequality between different social and economic groups | Implementation of the National Rural Health Program | Georgia  (Gamkrelide, Atun et al. 2002, Gotsadze, Zoidze et al. 2005) |
| Creating a platform for HIPPA^11^ implementation, health insurance liability law and liability  Increased insurance coverage | Approval of the accessible public health law  Adoption of Law HITECH ^10^  Adoption of the Law on Patient Protection and Cheap Health Care | Better relationships between patients and the care team  Reducing health care costs  Provide geographic-based services, relative market penetration of managed care  Population density  Lots of people without coverage | Providing services by health care organizations (HMO)^7^ in various forms, relationships and organizational models  NCQA^8^ National Committee for PCMH^9^ Quality Assurance Program  Patient-centred medical home  Patient-centred medical home | America  (Blumenthal and Dixon 2012, Lankarani 2012, O’Mahen and Petersen 2021) |
| Development of social welfare organizations  The stability of the transformation and leadership team  Lack of full realization of HSR^6^ goals due to gender and socio-economic differences | Full stewardship of Ministry Health  The Adoption of a comprehensive health transformation program with a flexible approach  Political commitment  Obtaining the consent of local political stakeholders | Expanding access to PHC  Increasing outpatient visits  Reducing screening and prevention programs  Lack of uniform improvement of quality and productivity of care among districts and regions | Creating a family doctor plan  Assignment of services to semi-governmental organizations and municipalities | Turkey  (Tatar and Kanavos 2006, Yasar 2011, Hone, Gurol-Urganci et al. 2017) |
| Attracting volunteers to participate in HSR  Lack of coordination and motivation  Health level differences between different SES^4^ groups | Interregional cooperation in HSR^6^  Decentralization in HSR^6^ | Increasing the use of services in rural areas  Improving population health  Insufficient quality of health centre services  More use of treatment services | Increasing population health coverage  Emplacing on outpatient care | Philippines (Obermann, Jowett et al. 2008) |
| Improving quality and access to services | Decentralization and transition of health care organization in each region to municipalities | Nursing homes, Baby day-care, social assistance, primary education | Providing social services | Finland  (Saarivirta, Consoli et al. 2012, Tynkkynen, Chydenius et al. 2016) |
| Increased access to health care  Increasing justice in service distribution | Adopting of the CatSalut ^12^ Law on Health Care Management, Planning, Service Purchasing and Evaluation | Increasing morbidity and preventive measures  Improving therapeutic aspects such as time and technical quality of treatment  Reducing the use of hospital services | Providing comprehensive multi-disciplinary team services including family physicians, nurses, midwives, paediatricians and others, under the supervision of a physician coordinator | Spain  (Larizgoitia and Starfield 1997) |
|  | Public health management reform | Increase immunization coverage | Creating a referral system for hospitalized patients  Public Health Focus on Prevention Programs | Kosovo  (Buwa and Vuori 2006, Percival and Sondorp 2010) |
| Improving public trust  Ability to freely select service providers by the population | Create a competition between private and public providers in the primary health care sector | Improving access to family physicians  Increasing referrals to physicians | Freedom of establishment and service by the private sector | Sweden  (Spak and Andersson 2008, Forsberg 2018) |
| Compensation for the deficit of family doctors  Increasing access to health care | Legal approval for the presence of internal medicine specialists and Pediatricians in the health care system |  | Strengthen the family doctor | Poland  (Mokrzycka, Kowalska-Bobko et al. 2016) |
| Expanding coordination in the actions of the whole country with the state and local levels and creating a coordinated approach to primary care planning and service delivery  Creating complex interactions between governmental, private and non-governmental organizations | Approving the National Strategic Framework for Strengthening Primary Health Care  Change in Government Focus: Setting up a central government bilateral program with each state  Changes in Processes: Labor Force Assessment - Clinical Leadership - Focused and Evidence-Based Decision Making - Reporting - Critical Assessment  Structural change: Establishment of a new primary health care organization MLs ^13^ (PHCO^14^ Evolution) - Establishment of the National Health Service Office - Australian Health and Safety Quality Commission - Local Hospital Network | Comprehensive health and social care include:  Care, prevention services, health promotion, social development  Attract social participation  Reduce hospitalization  Reduce health inequalities  Reduce mortality | Customer-centric care  Provide care at home and in the community  Developing partnerships between primary care physicians and local social determinants of health  Effective management of chronic disease | Australia  (Baum, Freeman et al. 2013, Baum, Freeman et al. 2016) |
|  | Municipalities play an important role in designing public health services  Most people use the public health department | Permission to provide services by the non-governmental sector  Strengthen the public sector while having a private sector | Choosing and changing your doctor freely  Continuity of care, family physician and efficiency in health care  Primary patient care by nurses and specialized care by a family physician  Taking care of chronic controlled patients by nurses  Taking care of sick, older people in nursing homes | Denmark  (Setlhare 2016) |
| Improving the infrastructure of health centres  Improving the monitoring of providers | Ethiopian Millennium Rural Plan (EMRI)^15^  Improving social participation and gaining the support of political and religious leaders | Providing system-based services  Improving the supply chain of services | Increasing and improving primary PHCU health care units  Establishing a referral system between health posts and health centres | Ethiopia  (Bradley, Byam et al. 2012) |
| Freedom to register and choose family doctors  Increasing the efficiency of health care  Improving quality of health care | Open registration program | Free primary health care to the general population | Further attention to primary health care (PHC) | Armenia  (Grigoryan 2005)  (ASTP)^16^  2015-2005 |
| Increasing authority and define diverse roles for nurses in primary health care | Increasing the understanding and participation of the local population in improving health  Review of nurses' duties | Changing from providing acute services to primary health care  Improving access, especially for residents of remote areas | The family-centred primary health care model focuses on the family physician and the collaboration of family health nurses | Commonwealth of Independent States of Central Asia (Parfitt 2009)  Tajikistan, Uzbekistan,  Azerbaijan, Georgia, Kyrgyzstan |
|  |  | Registration of the entire population with family physicians  Acceptance of the new health care system by the people | Elimination of monopoly on the provision of primary health care services by health centres | Slovenian (Vab 1995) |
| Designing of relevant health units for each local level following the policies of the Ministry of Health of the Central Government | Decentralization: Adoption of the law in 1997  Organizational adjustment programs | Improving access of health care | Providing services by local governments  Using private and public sector providers | Uganda  (Tashobya 2004) |
| Significant reduction in PHC "bypass" features  Improving service availability | Central policy-making and capacity building for organizing and managing, and providing services at the regional level  Improving relationships between people and PHC presentation units  Investing t in research on PHC reforms | Increasing the percentage of customers that use PHC facilities | Integration of PHC services, especially reproductive health and family planning services  Developing clinical guidelines for acceptance and adherence to quality standards | Albania (Hotchkiss, Piccinino et al. 2005) |
| Revival of primary health care that approved by PAHO^18^ | Development of legal frameworks  Approving the implementation of compatible care models with the social and cultural characteristics of the natives by recognizing their knowledge, methods and health resources | Implementation of care models reflecting the social and cultural characteristics of the population, community resources and epidemiological | Latin American Social Medicine LASM^17^  Empowering local communities  Focusing on the social determinants of health | South America  (Acosta Ramírez, Giovanella et al. 2016) |
| Increasing private sector participation in health care | Legislative reforms  Decentralization  Privatization | Free choice of physician  Reducing access to health care and increasing inequality in health care use | Strengthen the position of family physicians | Romanian  (Bara, Van den Heuvel et al. 2002) |
| Strengthen the structures needed for primary health care  Expand primary care cooperation with HCN ^22^centres and services internationally  Strengthen the decision-making process about health technologies | Organizational and administrative changes  Network definition and  TEIAS^19^ (Health Complex)  Establishment of Family Support Centers in Brazil NASF ^20^(2008)  Implement a decentralization policy  Adoption of the Law on HTA ^21^by the Federal 2011 | Expanding access  Appropriate relationship between citizens and professionals  Expanding the scope of primary care | Accreditation of services  Standardization of health methods and activities for all units  Development and simplification of care  Coordination of care and accountability  -Clinical governance | Brazil  (Almeida, Travassos et al. 2000, Kuchenbecker and Polanczyk 2012, Soranz, Pinto et al. 2016) |
| Changing the model of city-centred care to province-centered canton based)  The use of NGOs^24^ (Fami Foundation) as a facilitator of service delivery | Approving of the implementation of the local and sustainable model of the integrated FM ^23^approach in health care  Reducing political resistance and attracting the cooperation of all stakeholders  Corrections in organizational procedures | Increasing access  Providing services based on need | Family physician-based corrections  Multifaceted and simultaneous interventions at different levels of the health system | Bosnia and Herzegovina (Atun, Kyratsis et al. 2007)  FaMI Project  2004-2007 |
| Increasing attention to the quality and satisfaction, | Establishing independent family physician groups  Establishing of the Association of Non-Governmental Primary Care Physicians  Evaluating the performance of providers in general | Improving the level of responsiveness of primary care providers | Increasing the authority and autonomy of physicians in activity  Free selection of primary care providers through open registration | Kazakhstan  (Organization and UNICEF 1997, Abzalova, Wickham et al. 1998) |
| Better performance in most indicators  Improved satisfaction of clients  Gain health  Elimination of regional inequality | Setting policies through monitoring on key indicators  Decentralization of governance  Focus on quality and results framework (QOF)^25^  Elimination of hierarchical organizational structure  Establishment of family health units (USF)^26^  Establishment of ACeS ^27^cluster health centres | Improving quality and participation and accountability  Timely, easy and convenient access to services  Access to oral services for children, pregnant women, the elderly, and certain groups of patients (patients with HIV / AIDS and oral cancer) | Providing team services  Clinical governance  Providing services in government centres  Providing custom medical and nursing care  Oral and dental care in health care  Providing dental charges | Portugal  (Szczygieł, Pinto Lima et al. 2011, Biscaia and Heleno 2017)  Improve oral care  2008-2017 |
| Encourage the participation and cooperation of a wide range of stakeholders, including patients, providers and policymakers | Establish health and service centres  (HSSCs) ^29^and strengthen primary health care  Delegation of some affairs and authority and independence of provincial and state  Follow quality improvement strategies  Gradual change  Forming a working group of key stakeholders called the Primary Care Advisory Committee (PHCAC)^30^ | Improving the quality of care  Reducing outpatient users  Providing comprehensive services by multi-disciplinary health teams with free  Patient registration courses  Accessing to specialized services in network clinics | Implementing new methods and models of primary health care  Family physician groups and network clinics  Participatory care based on patient FHT^28^ | Canada  (Tenbensel 2008, Oandasan, Conn et al. 2009, Strumpf, Levesque et al. 2012, Harris, Green et al. 2015) |
| Coherent planning  Primary care providers are often private | Re-focus from regional health organizations (RHA)^31^ to strategic health organizations  Private sector participation in primary care | Full population coverage  Health promotion | Performing primary health care  Providing all health services  Providing some social services  Concluding contracts with specialists and hospitals | England  (Blumenthal and Dixon 2012) |
| Insurance of all Lithuanian residents  Achieving mutual cooperation  Informing and involving the whole community in decision making  Expansion of private service delivery | Approving the establishment of the State and Regional Insurance Fund (TSF)^32^  Approving the establishment of the Family Medicine Institute  Reviewing and emphasizing the decentralization of the health care sector  Strengthening the private sector  -Determine the position for nurses | Increasing the population covered by family doctors  The performance of a goalkeeper  A more comprehensive range of services (childcare, postpartum and adult services) | Introducing the family doctor as a key figure in the reformed health care system  Focus on improving health and preventing disease | Lithuania  (Liseckienė 2009, Buivydiene, Starkiene et al. 2010) |
| Increasing Productivity  Improving access and quality | Decentralization ESY^33^  Separating the provider with the buyer and creating a competitive market  Establishment of Regional Health Systems  (PE.S.Ys)^34^ | Improving patient access and providing evidence-based care  Combining clinics with each affiliated fund and developing urban health centres | Definition of new primary health care services including home care, medical care centres, rehabilitation centres  Establishing a new cooperation relationship between the public and private sectors | Greece  (Tragakes and Polyzos 1998, Tountas, Karnaki et al. 2002) |
| Providing health care for uninsured people  Reducing inequalities  Access to quality health services | Establishing a law about the community-based primary health care network  Integration of social and health services | Covering the entire population  Focus from treatment to prevention  Providing comprehensive and continuous health care, disease prevention, health promotion, diagnosis, treatment, monitoring and care | Establish 100 local health units TOMY^35^  Reviewing the service package  Reference mechanism  Providing team services |  |
| Combining innovative methods  Use the skills and resources of different organizations | Approval of the integration of medical education in the Ministry of Health  Approval of using programs of private sector  Public–private partnership (PPP)^36^  Health Cooperative Project (HCP)^37^  Health Complex Model (HCM)^38^  Decentralization of administrative and regulatory activities  Approval of insurance coverage for villagers and nomads and implementation of family physician  Approval of the Health Transformation Plan in the field | Increasing coverage  Expansion of services  Improving physical, financial and even cultural access to health services | Integration of mental health programs  Integration of professional health program  Integration of oral health program  Establishment of urban health bases  Hiring health volunteers  Establishing a family doctor  Integrating management and reduce disaster risk  Purchasing services from the non-governmental sector for residents of suburban areas and informal settlements | Iran  (Shadpour 2006, Lankarani 2012, Esmailzadeh, Rajabi et al. 2013, Asaei 2014, Malekafzali 2014, Heshmati and Joulaei 2016, Doshmangir, Moshiri et al. 2020) |

**Note:** Abbreviations:

1. PHO: Primary Health Organization
2. DHB: District Health Boards
3. NCMS: New Rural Cooperative Medical Scheme
4. SES: Socio-economic status
5. AUGE: Universal Access with Explicit Guarantees
6. HSR: Health sector reforms
7. HMO: Health maintenance organization
8. NCQA: National Committee for Quality Assurance
9. PCMH: Patient-Centered Medical Home
10. HITECH: Health Information Technology for Economic and Clinical Health
11. HIPPA: Health Insurance Portability and Accountability Act
12. CatSalut: law on ordering health care in Catalonia
13. MLs: Local Medical
14. PHCO: Pacific Health Care Organization
15. EMRI: Ethiopia Millennium Rural Initiative
16. ASTP: Armenian Urban Transfer Project
17. LASM: Latin American Social Medicine
18. PAHO: Pan-American Health Organization
19. TEIAS: Integrated Territories of Healthcare
20. NASF: Family Health Support Center
21. HTA: Health technology assessment
22. HCN: Health Care Networks
23. FM: Family medicine
24. NGO: Non-Governmental Organization
25. QOF: Quality and Outcomes Framework
26. USF: Family Health Units
27. ACeS: Health Center Clusters
28. FHT: Interprofessional family health teams
29. HSSC: Health and Social Services Center
30. PHCAC: Primary Healthcare Access Coordinator
31. RHA: Regional Health Authorities
32. TSF: Territorial Sickness Funds
33. ESY: National Health System in Greece
34. PE.S.Ys: Periferiaka Systimata Ygias
35. TOMY: Topikes Monades Ygias
36. PPP: public-private partnership
37. HCP: Health Cooperative Project
38. HCM: Health Complex Model

**Appendix S5: Review of primary health care reforms in different countries (medical products, vaccines & technologies and information)**

| **Information system** | | **Medicines and technology** | | **References**  **Country** |
| --- | --- | --- | --- | --- |
| **Outcome/impacts** | **Intervention** | **Outcome/impacts** | **Intervention** |  |
|  | Creating PCOs^1^ information systems | Controlling the price growth of prescription drugs | Establishing competitive standards and "cost and productivity" to enter national support packages;  Government funding to provide medicine | New Zealand (Gauld 2001, Tenbensel 2008) |
| Improvement in health management | Implementation of China Health Information System targeting specific diseases | Ensuring access  and safety of essential medicines | Introducing a list of essential medications  Providing general guidelines for drug production, prescribing and pricing | China  (Xu, Wang et al. 2007, Tang, Meng et al. 2008, Yip and Hsiao 2009, Hu, Zou et al. 2011, Sun, Chai et al. 2014, Lin, Sun et al. 2015) |
| Continuous and effective monitoring | Upgrading the health information system |  |  | Mexico  (Frenk, González-Pier et al. 2006, van Weel, Turnbull et al. 2016) |
| Lack of comprehensive health information | Primary health information system | Increase the level of general prescription and reduce the use of branded drugs | Introducing a new system for pharmaceutical facilities (data is not collected and reported in a way that allows  the health needs of these population sub-groups to be defined | UAE (Koornneef, Robben et al. 2012, Koornneef, Robben et al. 2017) |
| Lack of comprehensive health information | Primary health information system |  |  | Georgia (Gamkrelide, Atun et al. 2002, Gotsadze, Zoidze et al. 2005) |
| Protecting health information  Accessing to electronic health records | Health information technology for economic and clinical health |  |  | America (Blumenthal and Dixon 2012, Lankarani 2012) |
| Continuous and effective monitoring | Upgrading the health information system |  | Assignment of technology evaluation to semi-governmental organizations and municipalities | Turkey  (Tatar and Kanavos 2006, Yasar 2011, Hone, Gurol-Urganci et al. 2017) |
| Poor information system | Creating a health information system (HIS)^2^ |  |  | Kosovo(Buwa and Vuori 2006, Percival and Sondorp 2010) |
| Increasing research capacity and help to PHC knowledge | Implementing research strategies, assessment and primary health development, i.e., PHCRED^3^ |  |  | Australia  (Baum, Freeman et al. 2013, Baum, Freeman et al. 2016) |
| Ensuring the safety records and using them to improve patient care | Effective information technology network connecting different parts of the health field with reliable and fast technology |  |  | Denmark  (Setlhare 2016) |
| Maximum use of software systems | Creating an integrated software system that generates granular data |  |  | Ethiopia (Bradley, Byam et al. 2012) |
| Recording patient records | Modern cost accounting software |  |  | Armenia (Grigoryan 2005)  (ASTP)^4^  2015-2005 |
|  |  |  | Reforming infrastructure and basic equipment provision to enhanced health service provision  For effective primary health care services | Commonwealth of Independent States of Central Asia (Parfitt 2009) |
| Increased use of health information  Enhanced use of data for decisions | Create a new health information system  Medical records audit | Facilitated service delivery | Purchase of new equipment | Albania (Hotchkiss, Piccinino et al. 2005) |
| Recording information | Creating information systems |  |  | South America  (Acosta Ramírez, Giovanella et al. 2016) |
| Monthly evaluation of indicators through electronic files | Commitment to creating an information and computer system  Promoting research in services | Decisions about new health technologies | Establish a new HTA National Board (CONITEC)^5^  Establishment of Information and Communication Department of New Health Technologies (TICs)^6^ | Brazil  (Almeida, Travassos et al. 2000, Kuchenbecker and Polanczyk 2012, Soranz, Pinto et al. 2016) |
|  |  | Facilitating service delivery | Reconstruction and equipping of physical infrastructure | Bosnia and Herzegovina  (Atun, Kyratsis et al. 2007) |
| Accurate evaluation of service providers | New information systems |  |  | Kazakhstan  (Organization and UNICEF 1997, Abzalova, Wickham et al. 1998) |
|  |  | Evaluation and improvement of the quality and effectiveness of prescribing drugs by physicians | Pharmaceutical Reform Project | Croatia  (Harvey, Kalanj et al. 2004) |
| Electronic services | Continuous networking  Using social networks | Update service delivery | Using of new technologies | Portugal  (Szczygieł, Pinto Lima et al. 2011, Biscaia and Heleno 2017) |
| Creating a database | Integration of electronic medical records | Increasing the use of information technology | Implementation of information technology to provide decision-makers with evidence-based information to guide healthcare policy, planning, and funding | Canada  (Tenbensel 2008, Oandasan, Conn et al. 2009, Strumpf, Levesque et al. 2012, Harris, Green et al. 2015) |
| Improving service quality assessment | Focus on updating electronic health records |  |  | England  (Blumenthal and Dixon 2012) |
| Initial registration of information | Creating an electronic database of medical records |  |  | Greece  (Tragakes and Polyzos 1998, Tountas, Karnaki et al. 2002) |
| Accessing the information easily  More careful in assessing people's health and how to serve the people | Establishment of electronic health record | Evaluate existing technologies | Formation of the Office of Health Technology Assessment in the Office of Technology Assessment,  Development of standards and health tariffs | Iran  (Shadpour 2006, Lankarani 2012, Esmailzadeh, Rajabi et al. 2013, Asaei 2014, Malekafzali 2014, Heshmati and Joulaei 2016, Doshmangir, Moshiri et al. 2020) |

Notes: Abbreviations:

1. PCO: Primary Care Organizations
2. HIS: Health information system
3. PHCRED: Primary Health Care Research, Evaluation and Development
4. ASTP: Armenian Urban Transfer Project
5. CONITEC: National Committee for Technology Incorporation
6. TICs: Information and Communication Technologies in Health
